# Reinforcement of Styrene Butadiene Rubber Employing Poly(isobornyl methacrylate) (PIBOMA) as High *T*_g_ Thermoplastic Polymer

**DOI:** 10.3390/polym13101626

**Published:** 2021-05-17

**Authors:** Abdullah Gunaydin, Clément Mugemana, Patrick Grysan, Carlos Eloy Federico, Reiner Dieden, Daniel F. Schmidt, Stephan Westermann, Marc Weydert, Alexander S. Shaplov

**Affiliations:** 1 Luxembourg Institute of Science and Technology (LIST), 5 Avenue des Hauts-Fourneaux, L-4362 Esch-sur-Alzette, Luxembourg; abdullah.gunaydin@list.lu (A.G.); clement.mugemana@list.lu (C.M.); patrick.grysan@list.lu (P.G.); carloseloy.federico@list.lu (C.E.F.); Reiner.Dieden@list.lu (R.D.); daniel.schmidt@list.lu (D.F.S.); stephan.westermann@list.lu (S.W.); 2 Department of Physics and Materials Science, University of Luxembourg, 2 Avenue de l’Université, L-4365 Esch-sur-Alzette, Luxembourg; 3 Goodyear Innovation Center Luxembourg, L-7750 Colmar-Berg, Luxembourg; marc_weydert@goodyear.com

**Keywords:** thermoplastic polymers, styrene-butadiene rubber, reinforcement, mechanical properties, thermal properties

## Abstract

A set of poly(isobornyl methacrylate)s (PIBOMA) having molar mass in the range of 26,000–283,000 g mol^−1^ was prepared either via RAFT process or using free radical polymerization. These linear polymers demonstrated high glass transition temperatures (*T*_g_ up to 201 °C) and thermal stability (*T*_onset_ up to 230 °C). They were further applied as reinforcing agents in the preparation of the vulcanized rubber compositions based on poly(styrene butadiene rubber) (SBR). The influence of the PIBOMA content and molar mass on the cure characteristics, rheological and mechanical properties of rubber compounds were studied in detail. Moving die rheometry revealed that all rubber compounds filled with PIBOMA demonstrated higher torque increase values Δ*S* in comparison with rubber compositions without filler, independent of PIBOMA content or molar mass, thus confirming its reinforcing effect. Reinforcement via PIBOMA addition was also observed for vulcanized rubbers in the viscoelastic region and the rubbery plateau, i.e. from −20 to 180 °C, by dynamic mechanical thermal analysis. Notably, while at temperatures above ~125 °C, ultra-high-molecular-weight polyethylene (UHMWPE) rapidly loses its ability to provide reinforcement due to softening/melting, all PIBOMA resins maintained their ability to reinforce rubber matrix up to 180 °C. For rubber compositions containing 20 phr of PIBOMA, both tensile strength and elongation at break decreased with increasing PIBOMA molecular weight. In summary, PIBOMA, with its outstanding high *T*_g_ among known poly(methacrylates), may be used in the preparation of advanced high-stiffness rubber compositions, where it provides reinforcement above 120 °C and gives properties appropriate for a range of applications.

## 1. Introduction

Natural and synthetic rubbers have been used in a wide range of applications such as tires, elevator belts, hoses, tubes, adhesives, gloves, gaskets, coatings, antivibrational flooring, etc. [1,2,3]. However, the majority of rubber is employed in the production of tires, where many attempts have been made to enhance the properties of vulcanized rubber compounds [4,5]. One possibility for improving the physical properties of vulcanized rubber compounds including stiffness, storage modulus, tear resistance and tensile strength is the incorporation of fillers into the rubber matrix prior to vulcanization [6,7,8,9,10,11,12,13]. The most well-known fillers, namely, carbon black and silica, represented major breakthroughs in reinforcement technology when introduced in 1920s and 1990s, respectively [14,15]. Carbon black had been employed to improve abrasion resistance and mechanical strength [16]. Subsequently, the demand for a reduced carbon footprint [17], the production of more environmentally friendly tires and the realization of improved performance led to the introduction of silica as a “green” filler in rubber compositions leading to enhanced wet grip and rolling resistance performance [18,19].

While increasing the content of inorganic filler leads to more pronounced reinforcing effects in vulcanized rubber compounds, it can also lead to unwanted increases in tire mass and hysteresis [20,21,22]. As an alternative to inorganic fillers, the tire industry explored a broad range of lower density polymeric fillers in rubber compositions [23], leading to reductions in tires mass and rolling resistance [24]. Many different polymers, including polystyrene [25], syndiotactic polystyrene [26], poly(methyl methacrylate) [27], isotactic polypropylene [28,29], polyurethanes, poly(ethylene terephthalate) [30], polyamides [31,32], polycarbonates [33], syndiotactic polybutadiene (PBD) [21,22,34], ultra-high-molecular-weight polyethylene (UHMWPE) [35,36,37] and poly(alkyl methacrylate)s [38], were studied as thermoplastic fillers to improve the properties of rubber compositions (Table 1). Some of the aforementioned polymers provided additional enhancements in properties as well, including durability, stiffness and elasticity, reduced hysteresis, etc. [39,40]. Among them, UHMWPE [36,37] and syndiotactic PBD [41] have seen industrial application in rubber compositions destined for use in tires. The use of UHMWPE in particular has allowed for significant improvements in the stiffness of tire rubbers. However, due to its low softening or melting point (*T*_m_) (Table 1), above ~125 °C, UHMWPE rapidly loses its ability to provide reinforcement, thus limiting its usefulness [23]. Another issue with UHMWPE is its poor processability and the resultant difficulties effectively compounding UHWMPE-reinforced rubber formulations, thereby limiting the desired reinforcement effects due to relatively poor dispersion. In contrast to UHMWPE, syndiotactic PBD provides reinforcement over a wider temperature range [41] due to its high *T*_m_ (Table 1). However, high melting syndiotactic PBD is only produced in the form of a masterbatch with cis-1,4-polybutadiene rubber (see for example [22] or www.ube.com (accessed on 12 May 2021) for UBEPOL VCR412 and VCR617 products) meaning its use necessitates increasing the cis-1,4-polybutadiene content in a given rubber compound, which may be undesirable.

**Scheme 1:**
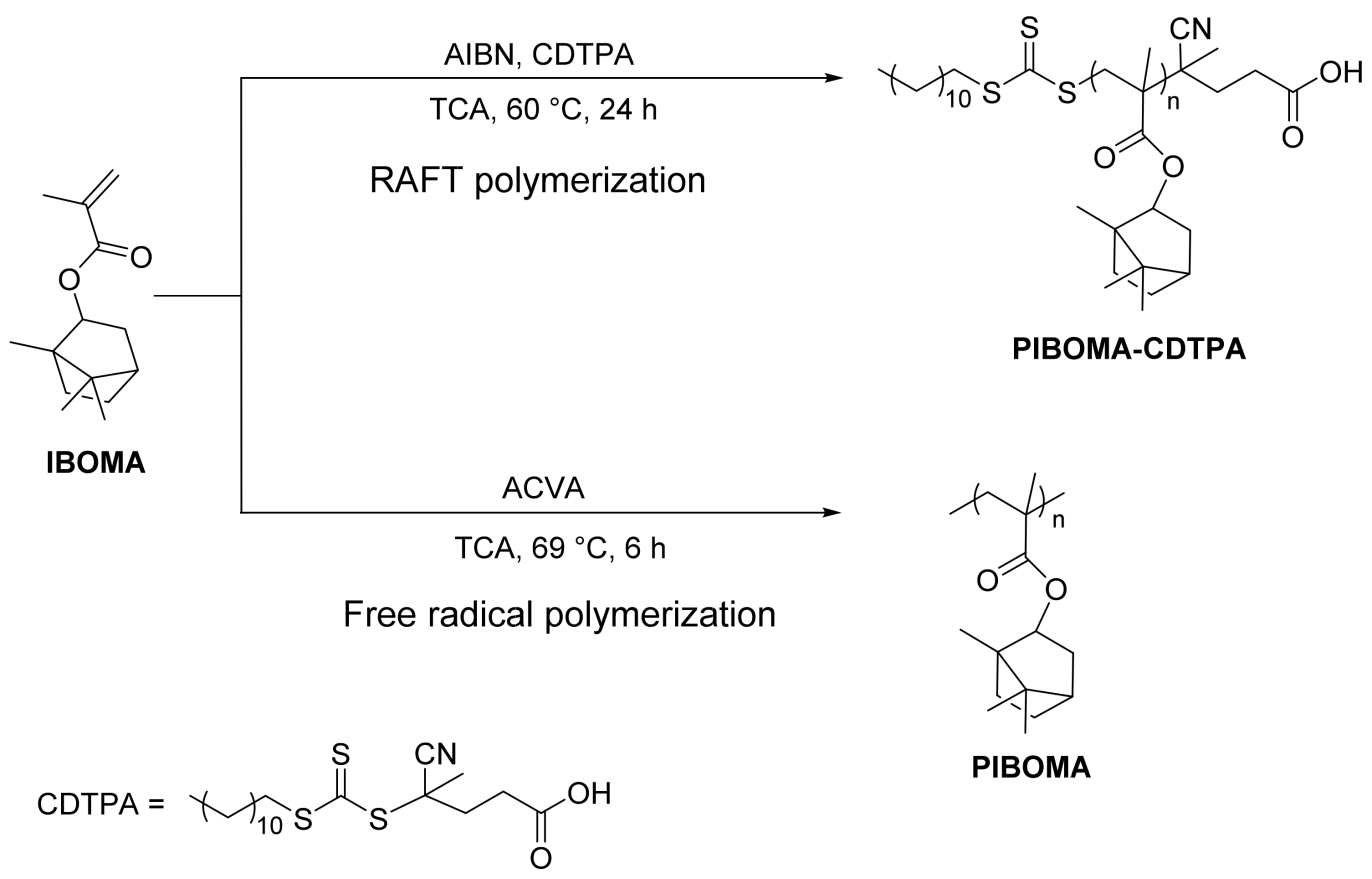
Schematic representation for synthesis of PIBOMA via free radical and RAFT polymerization.

As can be seen in Table 1, the majority of the thermoplastics investigated for rubber reinforcement possess thermal transitions at or below 125 °C, thus limiting their ability to reinforce vulcanized rubber compounds above this temperature. To overcome this disadvantage, in the present study, we report on the utilization of poly(isobornyl methacrylate) (PIBOMA) as a polymeric reinforcing filler for rubber. This polymer is attractive for several reasons. First, the bicyclic structure of the IBOMA monomer gives rise to methacrylate polymers with enhanced thermal stability and outstanding heat-resistance with glass transition temperatures *T*_g_ > 191 °C [42]. Second, IBOMA is a partially bio-based monomer coming from pine resin [43] and available at the ton scale. In this context, PIBOMA was prepared by two methods—free radical and reversible addition-fragmentation chain-transfer (RAFT) polymerization—with a variety of molecular weights ranging from 26,000 to 283,000 g mol^−1^ (Scheme 1) and further blended with styrene butadiene rubber (SBR). The investigation of the influence of the molar mass and content of PIBOMA on the properties of the resultant vulcanized rubber compounds allowed for the creation of advanced high-stiffness rubber compositions, capable of sustained mechanical performance well beyond 125 °C.

## 2. Materials and Methods

### 2.1. Materials

Solution-Styrene Butadiene Rubber (S-SBR) (Sprintan SLR-4602-Schkopau, Trinseo BVBA, Schkopau, Germany, composition: 21 wt% of styrene and 79 wt% of butadiene, vinyl content: 62 wt% (based on 1,4-butadiene); measured in this study: *M*_n_ = 243,000 g mol^−1^, *M*_w_/*M*_n_ = 1.6, *T*_g_ = −25 °C), ultra-high molecular weight polyethylene (UHMWPE, GUR 4120, Ticona, Sulzbach, Germany, *M*_n_= 4.7 × 10^6^ g mol^−1^), carbon black (CORAX N326 ASTM Carbon Black, Orion Engineered Carbons, Frankfurt, Germany, 78 m^2^ g^−1^—Nitrogen surface area in accordance with the requirements of ASTM D1765), diphenyl guanidine (97%, Draslovka a.s., Kolin, Czech Republic), oiled sulphur (98.5%, Siarkopol, Tarnobrzeg, Poland), stearic acid (95%, Aldrich, Darmstadt, Germany), *N*-(1,3-dimethylbutyl)-*N*′-phenyl-*p*-phenylenediamine (6PPD, anti-oxidant, 98%, ABCR, Karlsruhe, Germany), zinc oxide (99%, Everzinc, Liège, Belgium), treated distillate aromatic extracted oil (TDAE oil, Tudalen SX 500, Hansen & Rosenthal Gruppe, Hamburg, Germany), *N*-cyclohexyl-2-benzothiazolesulfenamide (CBS, 98+%, Duslo A.S, Šaľa, Slovakia), 4-methoxyphenol (99%, Aldrich, Darmstadt, Germany), *N*,*N*-dimethylformamide (DMF, anhydrous, 99.8%, Acros, Geel, Belgium), 4-cyano-4-[(dodecylsulfanylthiocarbonyl)sulfanyl]pentanoic acid (CDTPA, 97%, Aldrich, Darmstadt, Germany), 1,1′-azobis(cyclohexanecarbonitrile) (ACHN, 98%, Aldrich, Darmstadt, Germany) and 2,2′-azobis(*N*-butyl-2-methylpropionamide) (Vam-110, Wako, Japan), wax1 (31% *n*-paraffine, 69% *i*-paraffine, OK1887, Paramelt BV, Heerhugowaard, Netherlands) and wax2 (75% *n*-paraffine, 25% *i*-paraffine, OK1950H, Paramelt BV, Heerhugowaard, The Netherlands) were used without further purification. Tetrahydrofuran (THF), cyclohexane and toluene (all of SLR grade, Acros, Geel, Belgium) were purified through the SPS solvent purification system (MBraun, Garching, Germany, alumina and 4 Å sieves columns). 1,1,2-Trichloroethane (TCA, 97%, Aldrich, Darmstadt, Germany) and 1-methylnaphthalene (95%, Aldrich, Darmstadt, Germany) were purified by distillation over CaH_2_. Isobornyl methacrylate (IBOMA, VISIOMER^®^ Terra IBOMA, 99%, EVONIK Resource Efficiency GMBH, Essen, Germany) was distilled over CaH_2_ under reduced pressure (bp = 120–125 °C/1 mm Hg) prior to use. 2,2′-Azobis(2-methylpropionitrile) (AIBN, 98%, Aldrich, Darmstadt, Germany) and 4,4′-azobis(4-cyanovaleric acid) (ACVA, ≥98.0%, Aldrich, Darmstadt, Germany) were crystallized from methanol before utilization.

### 2.2. Measurements

NMR spectra were recorded on AMX-600 spectrometer (Bruker, Rheinstetten, Germany) at 25 °C in the indicated deuterated solvents over a range of −1 to 20 ppm (^1^H) and −20 to 220 ppm (^13^C) and are listed in ppm. The signal corresponding to the residual protons of the deuterated solvent was used as an internal standard for ^1^H and ^13^C NMR. Signal assignment was performed using 2D NMR techniques: heteronuclear single quantum coherence (HSQC), heteronuclear multiple bond correlation (HMBC), H-H correlation spectroscopy (H-H COSY). IR spectra were acquired on Brucker Tensor 27 Fourier IR-spectrometer (Brucker, Ettlingen, Germany) using ATR technology (128 scans, resolution is 2 cm^−1^) and Spectragryph optical spectroscopy software [44].

A 1260 Infinity II gel permeation chromatograph (GPC, Agilent Technologies, Santa Clara, CA, USA) was used to determine *M*_n_, *M*_w_ and PDI of the polymers. The chromatograph was equipped with integrated IR detector, a PLgel 5 mm MIXED-C, PLgel 5 mm MIXED-D columns and a PLgel guard column (Agilent Technologies, Santa Clara, CA, USA). THF was used as an eluent with a flow rate of 1.0 mL min^−1^ at 40 °C. Polymethylmethacrylate (Agilent Technologies, Santa Clara, CA, USA, Mp = 500 − 1500 × 10^3^ g mol^−1^) and polystyrene standards (Agilent Technologies, Santa Clara, CA, USA, Mp = 162 − 1500 × 10^3^ g mol^−1^) were used to perform calibration for the PIBOMA and SBR studies, respectively.

Thermal gravimetric analysis (TGA) was carried out in air on a TGA2 STARe System (Mettler Toledo, Zurich, Switzerland), applying a heating rate of 5 °C min^−1^. The onset weight loss temperature (*T*_onset_) was determined as the point in the TGA curve at which a significant deviation from the horizontal was observed. The resulting temperature was then rounded to the nearest 5 °C. Thermal mechanical analysis (TMA) of PIBOMA samples was performed under inert atmosphere (He) using a DIL 402 select Expedis dilatometer (NETZSCH, Selb, Germany) at a heating rate of 5 °C min^−1^ and a constant load of 0.3 N. Dynamic Mechanical Thermal Analysis (DMTA) measurements were carried out for cured compounds on bars (typically length × width × thickness = 20 × 6 × 2 (mm)) with a DMA 242 C model (Netzsch, Selb, Germany) operating in tension mode (strain between 0.05 and 0.07%, pretension: 10^−2^ N). Experiments were performed at 1 Hz frequency with a heating rate of 2 °C min^−1^ from −180 to 180 °C. The set up provided the storage and loss moduli (E′ and E″). The damping parameter or loss factor (tan δ) was defined as the ratio tan δ = E″/E′.

Atomic force microscopy (AFM) images were recorded with MFP-3D Infinity microscope (Asylum Research/Oxford Instruments, Abingdon, UK) in tapping mode (25 °C, in air). AC160TS-R3 (Olympus) cantilevers were applied with a stiffness of 26 N m^−1^ and resonance frequency of 300 KHz. The domain periodicity was evaluated from three different 1 × 1 μm^2^ images. On each image, two profiles were taken and for each, the distance over ten consecutive periods was recorded. The images were recorded in the so-called ‘soft tapping mode’, to avoid deformation and indentation of the polymer surface by the tip. All the images were collected with the maximum available number of pixels (512) in each direction. The general procedure for the preparation of the samples for AFM was as follows: cryo-cut of rubber was performed at −120 °C using N_2_ as a cryogenic liquid and a LEICA EM UC6 ultramicrotome (Leica Microsystems, Wetzlar, Germany) as cutting instrument. The blocks with a typical size (length × width × thickness = 10 × 5 × 5 (mm)) were trimmed to obtained a top surface of 500 × 200 microns which was gently polished with a diamond knife for further AFM analysis.

Mechanical properties of vulcanized rubber compounds, i.e., σ_t_—tensile strength (kPa), E_t_—tensile modulus (MPa) and ε—elongation (%) were measured at room temperature using a universal test machine Instron 5967 (Norwood, MA, USA) equipped with a load cell of 30 kN. The measurements were achieved at a cross-head speed of 5 mm/min. Before tests, samples were dumbbell-shaped by punching die machine according to ASTM standard D638-14 (Figure S8, Supplementary Materials) and stored during 48 h in the environmental conditions of measurement. At least 5 specimens were tested per reference.

Structural changes due to mechanical loading were visualized by micro-computed X-ray tomography (µCT). Post-mortem analysis of the fractured tensile samples was carried out with an EasyTom 160 (RX Solutions, Chavanod, France). Images were acquired at a tube voltage of 45 kV, current of 80 µA and a power of 3.6 W. These conditions provided a good phase contrast between the thermoplastic particles and the rubber matrix. Images were taken at 2 frames per second, using 5 average frames per projection. During the acquisition, samples were rotated 360° at angular steps of 0.25° while a flat panel recorded each projection. Source-to-object-distance (SOD) and source-to-detector-distance (SDD) were set to 10 mm and 210 mm, respectively, keeping a constant voxel size of 6 µm. The 3D volume reconstruction was carried out with the Xact64 software (RX Solutions, Chavanod, France), which enabled us to perform the inherent geometrical corrections, ring artefact removal and contrast enhancing.

### 2.3. Synthetic Procedures

#### 2.3.1. Synthesis of Poly(isobornyl methacrylate) via Reversible Addition−Fragmentation Chain-Transfer (RAFT) Polymerization

RAFT polymerization was performed to prepare a set of PIBOMAs with the molar mass varied from 20,000 to 60,000 g mol^−1^ (Table 2, PIBOMA 26K–PIBOMA 59 K). Typical polymerization procedure is given below by an example of PIBOMA40K synthesis.

IBOMA (20.00 g, 89 mmol), CDTPA (0.1009 g, 0.250 mmol) and AIBN (0.0103 g, 0.063 mmol, CDTPA:AIBN = 4:1 by mol) were dissolved in 14 mL of TCA in the Schlenk flask. The solution was deoxygenated by three freeze-pump-thaw cycles and sealed under inert atmosphere (Ar). The further synthesis was conducted at 60 °C for 120 h. Polymerization was quenched by the addition of 4-methoxyphenol (inhibitor), and the reaction viscous solution was diluted with dichloromethane. The resultant polymer containing the RAFT agent residue was isolated by double precipitation into the excess of methanol and dried at 55 °C/1 mm Hg for 12 h. Yield: 16.0 g (80%).

**Scheme 2:**
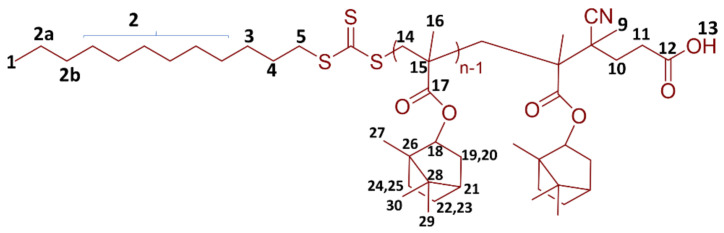
Schematic representation PIBOMA macromolecule with numbering for NMR assignment.

The assignment of ^1^H and ^13^C NMR was performed using numbering depicted in Scheme 2. ^1^H NMR (600.2 MHz, CDCl_3_): δ = 4.62–4.16 (br. m, 1H, H18), 2.4–1.6 (br. m, 5H, H14,H19,H21,H22), 1.54 (br. m, 1H, H24), 1.25 (<0.1H, m, Alk-2), 1.02–0.98 (br. m, 2H, H23,H25), 1.10–0.90 (br. m, 6H, H16, H27), 0.90–0.60 (br. m, 6H, H29,H30); ^13^C NMR (150.9 MHz, CDCl_3_): δ = 177.4, 176.5 (br. m, CO, C17), 83.1 (C18), 55.1 (br. m, C14), 44.9 (C26), 47.1 (C28), 45.3 (C21), 38.5 (C19), 37.5 (CH_2_S = Alk-5), 35.1 (CH_2_-CCN, C10), 34.5 (C24), 29.8 (Alk-2b), 27.2 (C22), 26.8 (CH_2_–COOH, C11), 29.5 (Alk-2), 25.1 (CH_3_CCN, C9), 24.2 (Alk-2a), 20.3 (C27 & C29), 19.2–17.1 (br. m, C16), 14.3 (Alk-1); IR (ATR-mode): 2952 (s, CH), 2877 (m, CH), 1722 (vs, C=O), 1472 (m, CH_3_), 1454 (s, CH_2_), 1390 (m, α-CH3), 1370 (m), 1311 (w), 1295 (w), 1237 m, asC–C), 1146 (vs, sC–O–C), 1109 (s), 1082 (m), 1051 (vs, CH_2_), 1002 (s), 969 (s), 944 (m), 911 (w), 886 (m), 860 (w), 843 (m), 789 (w), 753 (m, α-CH_3_), 704 (w), 629 (w), 585 (w), 547 (w), 515 (m). cm^−1^; *M*_n theo_ = 80,000 g mol^−1^; *M*_n SEC_ = 46,500 g mol^−1^; *M*_w_/*M*_n_ = 1.2 (SEC); *T*_g_ = 197 °C (TMA); *T*_onset_ = 230 °C (TGA).

For kinetics studies the polymerization was performed as described above, however, with the exception that 1-methylnaphthalene (0.10 g, 0.70 mmol) was added in the reaction solution as internal reference for the determination of the conversion. Aliquots were removed from the reaction flask under argon atmosphere using a syringe at predetermined time intervals throughout the polymerization. ^1^H NMR spectroscopy in CDCl_3_ and gel permeation chromatography (GPC) in THF were used to determine the monomer conversion and the product molecular weights, respectively. Monomer conversion was calculated by relative integration of vinyl protons of residual IBOMA monomer and protons of the methyl group from 1-methylnaphthalene (used as an internal reference).

#### 2.3.2. Synthesis of Poly(isobornyl methacrylate) via Free-Radical Polymerization

To overcome the limitations of RAFT polymerization and with the aim to prepare PIBOMA with *M*_n_ > 200,000 g mol^−1^, the free radical polymerization of IBOMA was performed. Typical polymerization procedure is given below by an example of PIBOMA283K synthesis:

IBOMA (20.0 g, 89 mmol) and Vam-110 (0.1000 g, 0.312 mmol, 0.5 wt%) were dissolved in 14 mL of TCA in the Schlenk flask. The solution was deoxygenated by three freeze-pump-thaw cycles and sealed under argon. Polymerization was carried out at 110 °C for 6 h. The reaction was quenched by the addition of 4-methoxyphenol (inhibitor) and the very viscous solution was diluted with dichloromethane. The resultant polymer was isolated by double precipitation from dichloromethane solution into the excess of methanol and dried at 70 °C/1 mm Hg for 12 h. Yield: 14.4 g (72%); *M*_n SEC_ = 283,000 g mol^−1^; *M*_w_/*M*_n_ = 1.9 (SEC); *T*_g_ = 199 °C (TMA); *T*_onset_ = 225 °C (TGA).

#### 2.3.3. Rubber Compounding

The compounding of SBR rubber and thermoplastics was performed in accordance with the procedure published previously [45], although with some minor modifications. A HAAKE PolyLab OS internal mixer (Thermo Scientific, Germany) was used to prepare all rubber compositions. Mixing was carried out in a Rheomix 600 chamber with a free volume of 85 cc, using two cam-type rotors and with a constant fill factor of 0.75. The mixing process was performed in two stages: (1) incorporation of filler, antioxidants, activators and waxes into the elastomer matrix; (2) addition of curatives and accelerators. The detailed formulation of each rubber composition is presented in Table 2.

In the first stage, also referred to as the non-productive (NP) stage, rubber; filler and other ingredients such as antioxidants, activators and waxes (Table 2, stage NP), were combined at an initial temperature of 80 °C with a rotor speed of 40 rpm. After 2 min of mixing the rotor speed was gradually increased to 60 rpm for 2 min and finally to 80 rpm for 7 min. During incorporation of filler and additives, the temperature was observed to rise to ~120–127 °C. Thereafter, the mixed batch was discharged from the internal mixer and taken to the laboratory-scale open two roll mill (Polymix 110L, Servitec Maschinenservice GmbH, Wustermark, Germany). It was passed through the roll mill six times using back and front rotor speeds of 24 and 32 rpm, respectively, and a roll gap of 2.0 mm. The temperature of the rolls was set to 50 °C. A relaxation time of ~1 h was given prior to the second stage of mixing.

In the second stage, also referred to as productive (PR) stage, the resultant rubber compounds were further mixed with curatives and accelerators (Table 2, stage PR) in the internal mixer at an initial temperature of 60 °C with a rotor speed of 40 rpm. The mixing was continued for 2 min, during which time the temperature was observed to rise to ~75–80 °C. In this mixing step, the temperature was not allowed to exceed 80 °C in order to avoid premature vulcanization. Finally, the mixed batch was discharged from the internal mixer and processed a second time using the open two roll mill (as described above).

#### 2.3.4. Cure Characterization

The curing characteristics of the rubber compounds were investigated using a moving die rheometer (MDR, MDR2000, Alpha Technologies, Hudson, OH, USA). The MDR was preheated at 150 °C for approximately 30 min. After that, the test was performed at 150 °C for 60 min with an oscillation of amplitude of 0.5° (~7% strain) and a frequency of 1.667 Hz. The optimum cure times (t_90_) were calculated from the curves of each compound as the time required for the torque to reach the value given by the Equation (1):

*S*(t_90_) = 0.9 (*S*_max_ − *S*_min_) + *S*_min_ (1)

where *S*_max_ is the maximum achieved torque, and *S*_min_ is the minimum torque on the curve.

The torque increase (Δ*S*) was determined as the difference between the *S*_max_ and *S*_min_ according to Equation (2):

Δ*S* = *S*_max_ − *S*_min_ (2)

#### 2.3.5. Vulcanization

The milled sheets were vulcanized in a stainless-steel mold having rectangular and square frames (length × width × thickness = 80 × 30 × 2 and 40 × 40 × 2 (mm), respectively). Vulcanization was carried out applying a hydraulic pressure of 150 bars using the LP-S-50 press (LabTech Engineering Co. Ltd. Samut Prakan, Thailand) for 18 min at 150 °C. The chosen cure time was for all crosslinked samples slightly above t_90_ calculated from the MDR curves. The final thickness of the cured samples varied in the range of 1.9–2.0 mm.

## 3. Results and Discussions

### 3.1. Synthesis of Poly(isobornyl methacrylate) (PIBOMA)

To examine the influence of the molecular weight of PIBOMA on the mechanical properties of rubber composition, the reversible addition−fragmentation chain-transfer polymerization (RAFT) technique was chosen for the synthesis of PIBOMA samples with precise molar masses [46]. 4-Cyano-4-[(dodecylsulfanylthiocarbonyl)sulfanyl]pentanoic acid (CDTPA) was selected as RAFT-agent as it was previously successfully used for RAFT polymerization of (meth)acrylic monomers allowing perfect control over reaction rate and polymer molar mass [47]. 1,1,2-Trichloroethane (TCA) was chosen as a high boiling analog from chlorinated family of solvents that have proven themselves to be a suitable media for successful RAFT polymerization [47].

**Figure 1:**
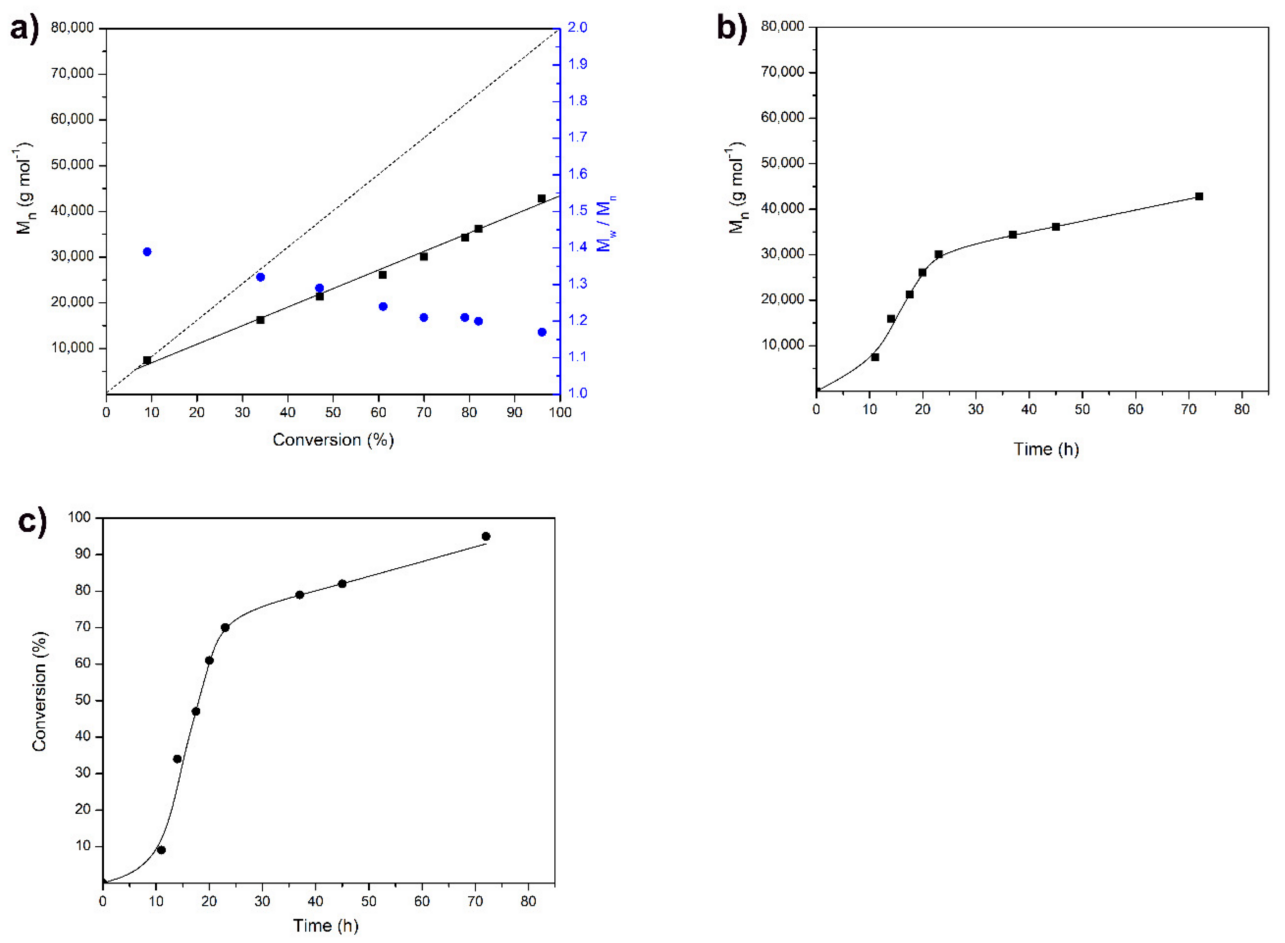
*M*_n_ vs. conversion (**a**), *M*_n_ vs. time (**b**) and conversion vs. time (**c**) kinetic plots for IBOMA RAFT polymerization (TCA as a solvent, CDTPA:AIBN = 4:1 by mol, [IBOMA] = 1.44 g mL^−1^ or 50 wt%, *M*_n theor_ = 50,000 g mol^−1^).

Initially, the study of PIBOMA’s RAFT polymerization was carried out by an example of CDTPA to monomer ratio set to target a molecular weight (*M*_n theo_) of 80,000 g mol^−1^ (Figure 1). The probes were taken from the reaction at different times, and each sample was analyzed by ^1^H NMR and GPC, to evaluate monomer conversion and polymer molecular weight, respectively. It was revealed that the almost quantitative conversion of the monomer (~96%) was obtained after 72 h. It was found that IBOMA is polymerizing at 60 °C in a controlled manner, i.e., a linear increase in number average molecular weight (*M*_n_) with the rise in conversion and satisfactory low dispersity indices *M*_w_/*M*_n_ < 1.35 (Figure 1a). At this, the experimental M_n_ values determined by GPC in THF were nearly half compared to the theoretically calculated ones (Figure 1b). This discrepancy can be probably explained by the steric hindrance of the bulky isobornyl group, thus lowering the reactivity of IBOMA monomer when homopolymerization takes place. However, when IBOMA was copolymerized via the RAFT method with other methacrylic monomers, such a deviation was not observed [48]. Another possible explanation can be connected with the utilization of PMMA standards for GPC calibration and some structural difference between PIBOMA and PMMA. In spite of the *M*_n_ values being lower than expected, the dispersity values (*M*_w_/*M*_n_) stayed satisfactorily low and were in the range 1.16–1.21 (Figure 1a and Figure S1; Supplementary Materials). More generally, it is observed that *M*_n_ vs. time dependence appears to follow a sigmoidal curve in this system (Figure 1b), with an apparent induction period of several hours prior to a rapid increase. Similar behavior has been reported in other controlled radical polymerizations involving sulfur-based RAFT agents [47]. Then, following approximately one day of reaction, the rate of increase of *M*_n_ slows significantly, which may be related to considerable increases in viscosity of the polymer solution.

**Figure 2:**
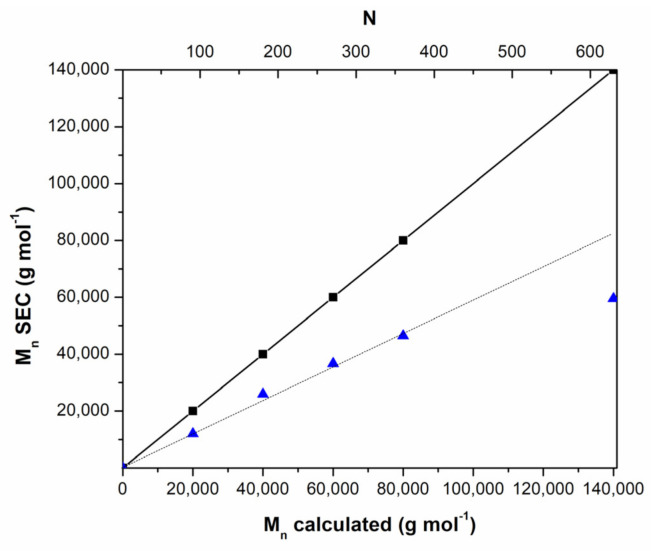
Correlation of experimental PIBOMA *M*_n_ (▲), values from RI-detection, SEC in THF at 40 °C) with calculated theoretical *M*_n_ (■) or calculated number of monomer equivalents N.

Once the optimal reaction conditions were established, a set of polymers with molecular weights in the range from 26,000 to 59,500 g mol^−1^ were obtained via RAFT polymerization of IBOMA (Table 3, entries 1–3 and Figure 2). In all cases the dispersity values were satisfactorily low ≤ 1.21. The linear increase of the determined M_n_ values plotted as a function of theoretically calculated ones (Figure 2) supports the control of polymerization until molar mass reaches ~50,000 g mol^−1^.

The obtained PIBOMA samples represented powder-like materials with yellowish color, the latter being due to the utilization of yellow CDTPA agent. Their structure was confirmed by ^1^H and ^13^C NMR and IR spectroscopy (Figures S2 and S3). ^1^H NMR shows three broad sets of signals at 4.62–4.16, 2.4–1.6 and 1.55–0.60 ppm attributed to the target polymer (Figure S2a). The residual protons for the end-groups from CDTPA RAFT agent were expected at 4.2, 3.7, 3.5 and 2.1–0.9 ppm, but cannot be seen in ^1^H NMR due to the overlap with the signals coming from main chain and side isobornyl group. The peaks of the vinyl group from IBOMA monomer (6.05 and 5.50 ppm) were absent, proving the purity of the obtained polymers. The broad signals at 2.39–1.64 and 1.00–0.84 ppm were assigned to CH_2_ and CH_3_ groups of the polymer backbone, respectively (Figure S2a). The isobornyl group was represented by peaks at 4.45–4.2, 2.33–1.69, 1.54, 1.07 and 0.94–0.62 ppm, correspondingly (Figure S2a). The detailed attribution of the carbon peaks was performed using 2D NMR techniques: heteronuclear single quantum coherence (HSQC), heteronuclear multiple bond correlation (HMBC) and H-H correlation spectroscopy (H-H COSY) and is shown in Figure S2b. FTIR spectra of PIBOMA is presented in Figure S3. The peaks at 2952 and 2877 cm^−1^ correspond to aliphatic CH stretching vibrations of the CH_3_ and CH_2_ groups. A sharp intense peak appearing at 1772 cm^−1^ is ascribed to the ester carbonyl group (C=O) stretching vibration. The bands at 1237 and 1146 cm^−1^ are assigned to asymmetric and symmetric C–O–C stretching vibrations of the ester groups, respectively (Figure S3). The two bands at 1390 and 753 cm^−1^ can be attributed to the α-methyl group vibrations. The vibrational peak appeared at 1472 cm^−1^ is associated with CH_3_ stretching vibrations, while the peaks at 1454 and 1051 cm^−1^ are assigned to CH_2_ scissoring vibration and CH_2_ skeletal stretching vibration in the isobornyl cycle.

Due to the limitations of the RAFT method for the controlled synthesis of high molar mass polymers (*M*_n_ > 80,000 g mol^−1^ [49]), the decision was made to enlarge the set of PIBOMA samples by one polymer with high molar mass prepared via free-radical polymerization (Table 3, entry 4). As in the case of RAFT polymerization, the study of a free-radical polymerization started with the determination of the optimum reaction conditions (Table S1). First, the influence of solvent was examined. The polymerization proceeded in solution in toluene, THF, cyclohexane and 1,1,2-trichloroethane (1,1,2-TCA), while in DMF precipitation of the polymer was observed (Table S1, entries 1–5). The utilization of DMF, toluene and cyclohexane provided only moderate yields below 45% (Table S1, entries 1, 2 and 4). At the same time IBOMA polymerization in THF and 1,1,2-TCA resulted in polymer formation with yields up to 74% (Table S1, entries 3 and 5). Among the tested solvents, the highest molecular weight of the polymer was achieved in cyclohexane (*M*_n_ = 151,800 g mol^−1^), while all other candidate solvents provided polymers with the similar molar masses varying in the range of 59,500–73,600 g mol^−1^ (Table S1, entries 4 and 1–3, 5). In the second step the effect of initiator was investigated (Table S1, entries 5–8). This study involved AIBN, ACHN, ACVA and Vam-110 azo-type radical initiators. It was found that the use of Vam-110 in 1,1,2-TCA leads to the synthesis of PIBOMA with the highest molar mass (82,900 g mol^−1^) and the highest yield (80%) (Table S1, entry 8). Finally, two different concentrations of Vam-110 initiator have been tried: 1.0 and 0.5 wt% (Table S1, entries 8–9). In addition, although the decrease in the initiator’s concentration leads to an insignificant decrease in polymer’s yield down to 70%, the molar mass of the obtained PIBOMA was tripled. Concluding this part on free-radical radical polymerization, the following reaction parameters providing the polymer having the highest molecular weight were found to be optimal: 1,1,2-TCA as solvent, temperature 100 °C, reaction duration 6 h, [IBOMA] = 50 wt% and [Vam-110] = 0.5 wt%. These conditions were used for the scale up of the reaction and allowed for the synthesis of PIBOMA with high molar mass (*M*_n_ = 283,000 g mol^−1^) and moderate dispersity (*M*_w_/*M*_n_ = 1.90).

### 3.2. Properties of PIBOMA

The solubility of the obtained PIBOMA samples was studied in a variety of solvents (Table S2). It was found that PIBOMA is soluble in toluene, some of the polar aprotic solvents (THF, diethyl ether, dimethylacetamide (DMAc)) and chlorinated solvents (dichloromethane (DCM), chloroform, 1,1,2-TCA) at ambient temperature. It was possible to dissolve these polymers in *N*,*N*-dimethylformamide (DMF) and cyclohexane upon heating to 80 °C. PIBOMA was found to be insoluble in acetonitrile, acetone, alcohols (MeOH, EtOH) and in *N*-methyl-2-pyrrolidone (NMP).

Polymer thermal properties were investigated by thermomechanical (TMA) and thermogravimetric analysis (TGA). According to TMA (Figure S4 and Table 3), the PIBOMA samples demonstrated high *T*_g_ varying in the range of 194–201 °C, and depending on their molar masses, they can be arranged in the following increasing order:

*T*_g_ PIBOMA 12 K (194 °C) < *T*_g_ PIBOMA 26 K (197 °C) = *T*_g_ PIBOMA 46 K (197 °C) ≤ *T*_g_ PIBOMA 283 K (199 °C) < *T*_g_ PIBOMA 59 K (201 °C).

It can be concluded, that in the studied range of molar masses the *T*_g_ of PIBOMA was found to be practically independent on its molecular weight.

The onset mass loss temperatures (*T*_onset_) of the neat PIBOMA samples evaluated by TGA (Figure S5) revealed their high thermal stability ranging from 220 to 230 °C. The *T*_onset_ values of neat PIBOMA samples decrease insignificantly following the order below with respect to the molecular weight of the polymers (Table 1):

*T*_onset_ PIBOMA 12 K (220 °C) < *T*_onset_ PIBOMA 26 K (225 °C) = *T*_onset_ PIBOMA 46 K (225 °C) = *T*_onset_ PIBOMA 283 K (225 °C) < *T*_onset_ PIBOMA 59 K (230 °C).

### 3.3. Compounding

The compounding of SBR rubber and thermoplastic was performed in accordance to the procedures published previously [45]. The detailed formulation of rubber compositions is presented in Table 2. All prepared compounds contained 50 phr of carbon black. The influence of PIBOMA’s molar mass on properties of rubber compositions was studied on samples where the content of thermoplastic was fixed at a level of 20 phr. Further on, the influence of the PIBOMA amount (in phr) on the characteristics of the filled vulcanized rubber compounds was investigated using the PIBOMA sample that showed the highest reinforcement on the previous step. Vulcanized rubber compounds containing thermopastic and carbon black fillers are abbreviated here as Thermoplastic name/CB (PIBOMA 20 K/CB, for example). The vulcanized rubber composition based solely on SBR and carbon black was used as a reference for comparison and is denoted here as SBR/CB rubber.

#### 3.3.1. MDR

**Figure 3:**
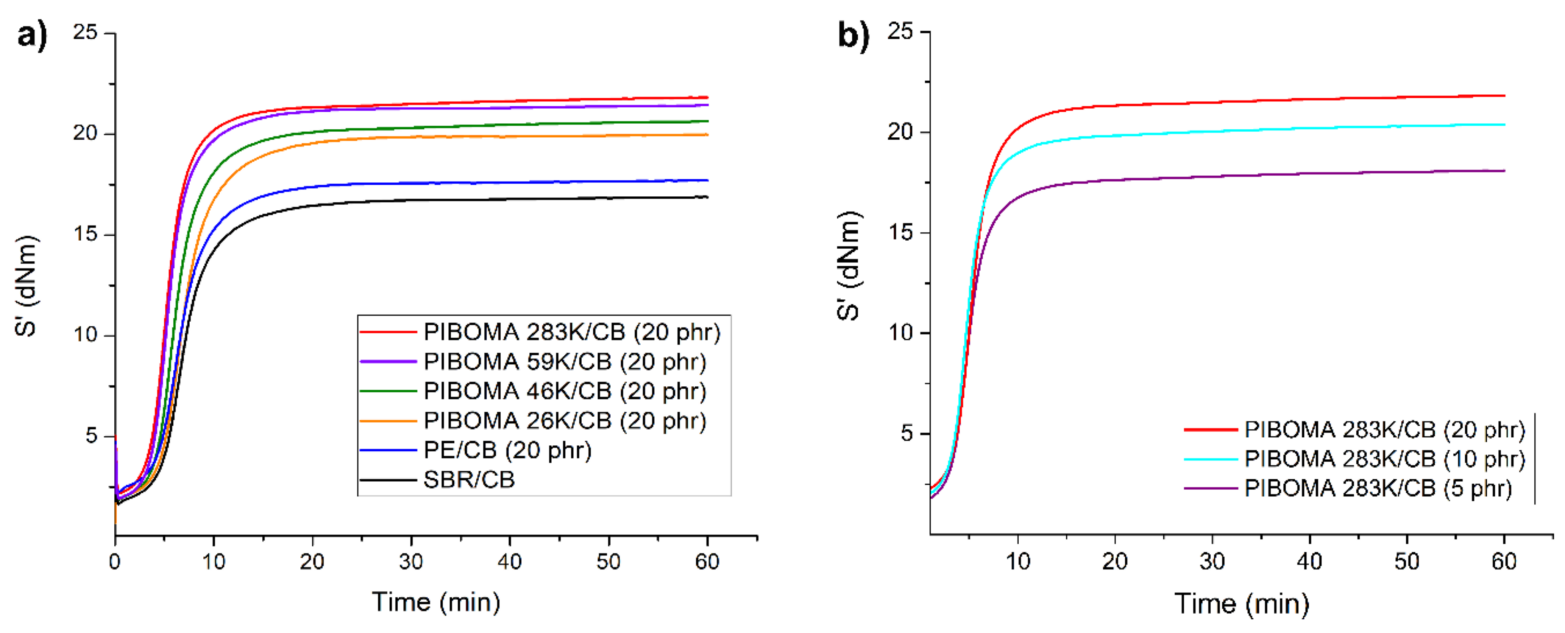
Rheographs of rubber compounds filled with 50 phr of CB and 20 phr of PIBOMA with variable molecular weights (**a**) and of those containing different amounts of PIBOMA 283 K (**b**).

The cure characteristics of rubber compounds filled with 50 phr of carbon black (CB) and 20 phr of PIBOMA samples having molecular weight in the range from 20,000 to 283,000 g mol^−1^ were investigated by moving die rheometry (MDR) (Table 3 and Figure 3a). The test of the rubber composition filled with 50 phr of CB and 20 phr of ultra-high molecular weight polyethylene (UHMWPE) was performed for comparison (Figure 3a). The torque increase values (Δ*S*) obtained from the difference between maximum torque (*S*_max_) and minimum torque (*S*_min_) were used for the estimation of the reinforcing effect. Relative to the CB control compound, all samples filled with CB and PIBOMA displayed higher Δ*S* values, irrespective of PIBOMA molecular weight, confirming the high temperature reinforcing effect of PIBOMA. In contrast, the sample filled with CB and UHMWPE displayed a Δ*S* value close to the CB control in accordance with the softening of UHMWPE at the MDR measurement temperature (150 °C). The increase in the PIBOMA’s molecular weight led to the rise in Δ*S* values of the respective rubber compositions (Figure 3a). The influence of the PIBOMA and its molar mass on the viscoelastic properties of vulcanized rubber compounds can be summarized in the following increasing order:

Δ*S* SBR/CB (15.2 dNm) < Δ*S* UHMWPE/CB (15.5 dNm) < Δ*S* PIBOMA 26 K/CB (18.1 dNm) < Δ*S* PIBOMA 46 K/CB (18.8 dNm) < Δ*S* PIBOMA 59 K/CB (19.5 dNm) < Δ*S* PIBOMA 283 K/CB (19.7 dNm).

The highest reinforcement was shown by a sample containing 20 phr of PIBOMA having *M*_n_ = 283,000 g mol^−1^, and thus, the PIBOMA 283 K was further selected to study the effect of thermoplastic content on the rheological properties of rubber compounds (Figure 3b). Comparing the torque increase values of rubber compositions filled with 5, 10 and 20 phr of PIBOMA 283 K, it is possible to arrange them in the following order:

Δ*S* PIBOMA 283 K 5 phr/CB (15.9 dNm) < Δ*S* PIBOMA 283 K 10 phr/CB (18.2 dNm) < Δ*S* PIBOMA 283 K 20 phr/CB (19.7 dNm).

Thus, the higher the content of PIBOMA 283 K was, the higher Δ*S* value was demonstrated by the respective rubber composition, indicating higher reinforcement.

#### 3.3.2. DMTA

**Figure 4:**
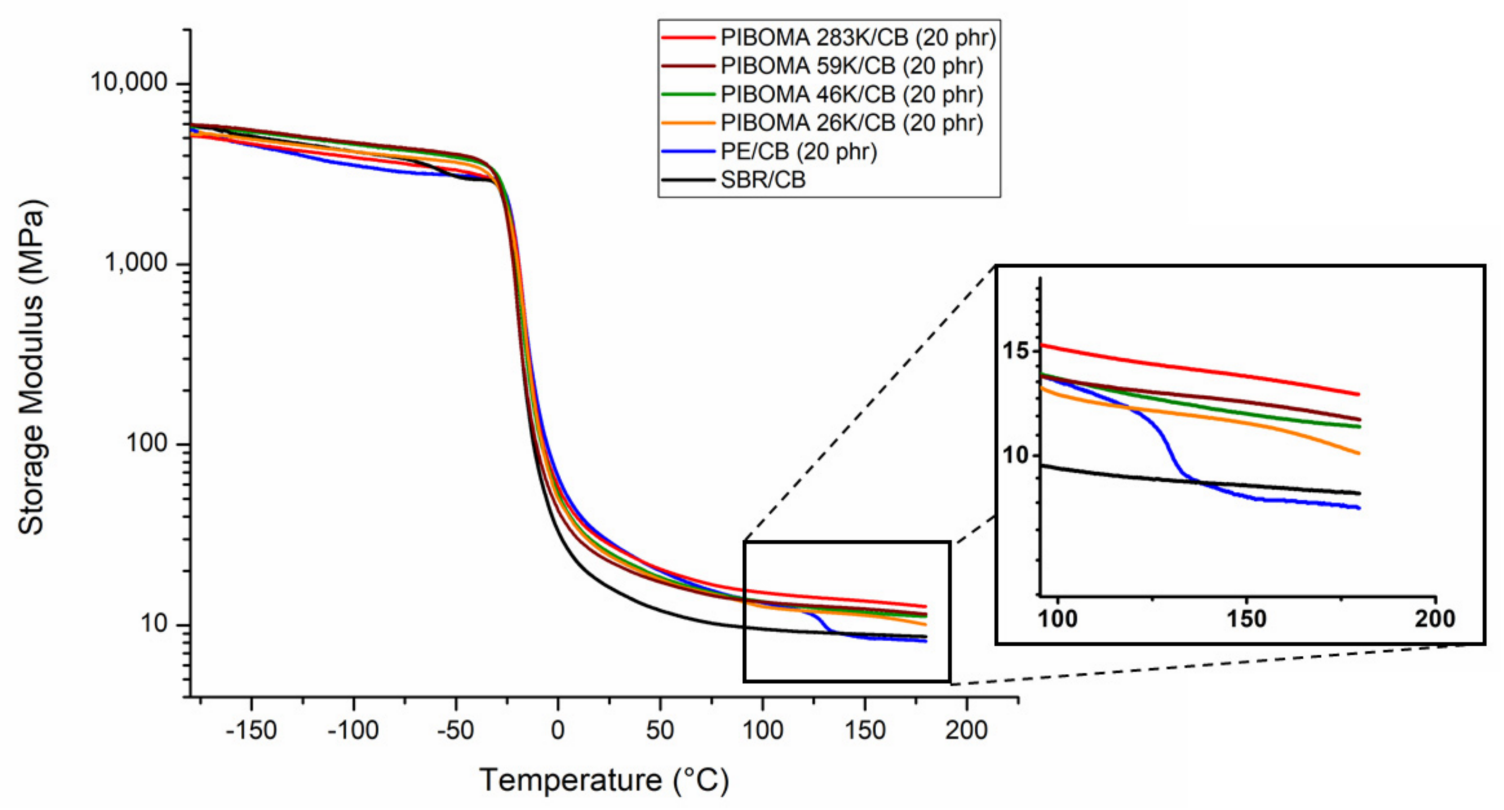
The storage modulus as a function of temperature for vulcanized rubber compounds containing 50 phr of CB and filled either with 20 phr of PIBOMA having various molecular weights or with 20 phr of UHMWPE.

**Figure 5:**
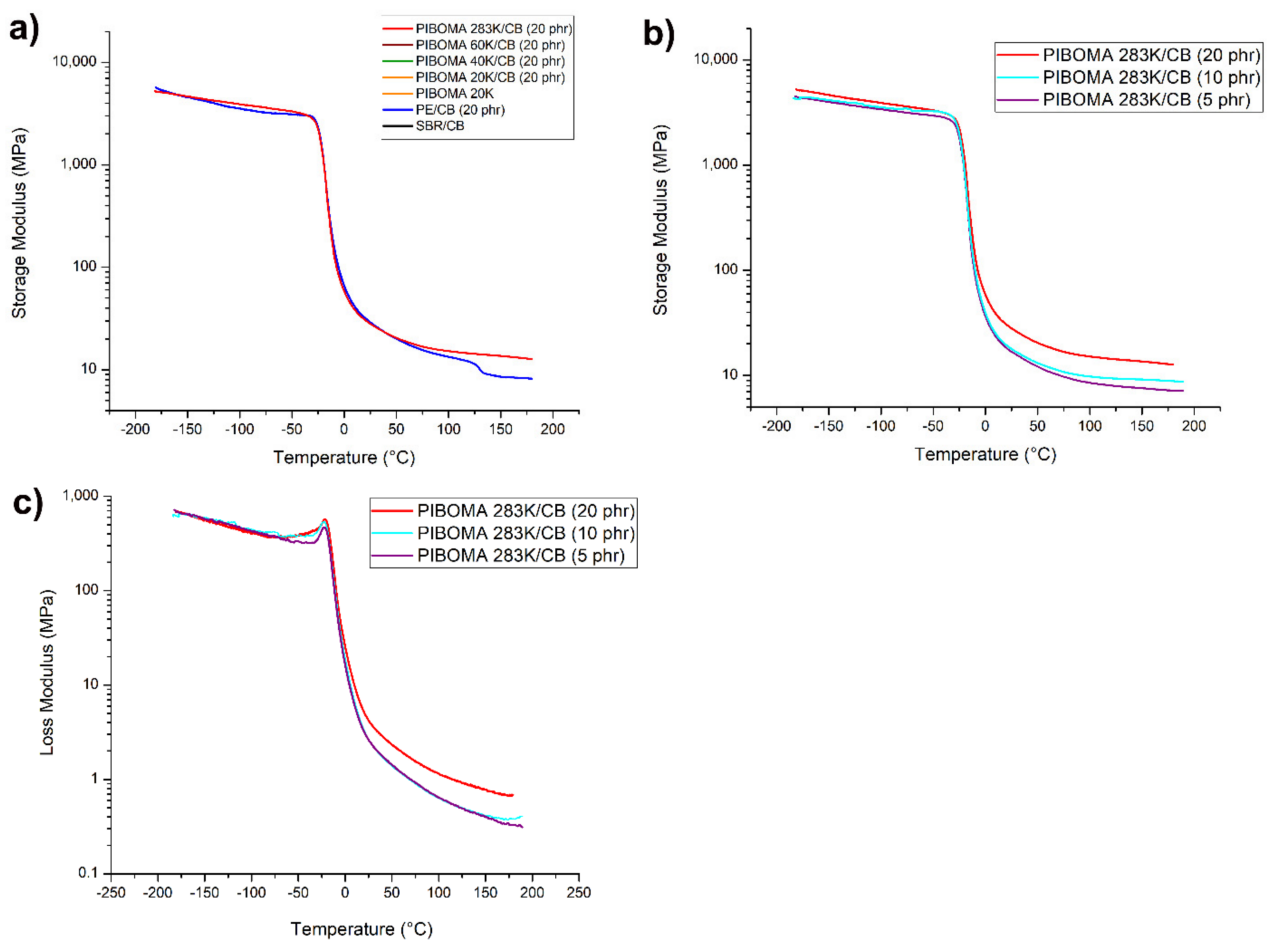
The storage modulus as a function of temperature for vulcanized rubber compounds containing 50 phr of CB and filled with 20 phr of PIBOMA 283 K and 20 phr of UHMWPE for comparison (**a**) and of those containing different amounts of PIBOMA 283 K (**b**). Loss modulus vs. temperature for vulcanized rubber compounds containing 50 phr of CB and filled with different amounts of PIBOMA 283 K (**c**).

The storage modulus of vulcanized rubber compounds filled with PIBOMA having molecular weights ranging between 20,000 and 283,000 g mol^−1^ and UHMWPE is presented in Figure 4 and Figure 5a. In the rubbery regime (>−15 °C), all thermoplastics (PIBOMA, PE) provided similar or higher storage moduli as compared to that of the SBR/CB control (Figure 4, black line), confirming thermoplastic reinforcement in all PIBOMA/CB and PE/CB vulcanized rubber compositions. Considering the PIBOMA reinforced compounds alone, it is observed that the increase in storage modulus vs. the SBR/CB control varies with molecular weight. In the glassy plateau, lower molecular weights give greater increases in storage moduli, while in the rubbery regime, higher molecular weights give larger increases in storage moduli. In the case of UHMWPE (Figure 4 and Figure 5a, blue lines), little or no reinforcement is observed in the glassy regime, with more significant reinforcement observed above the glass transition temperature (*T*_α_ more exactly). Comparing now these two polymer reinforcement systems, several observations can be made. From ~0–30 °C, PIBOMA 283 K provides competitive levels of reinforcement vs. UHMWPE (Figure 5a). In the range of ~30–125 °C, the reinforcing efficiency of the UHMWPE drops slightly, such that the lower molecular weights of PIBOMA become competitive at the higher end of this temperature range. Furthermore, PIBOMA 283 K exceeds the performance of UHMWPE throughout this range. Above ~125 °C, the UHMWPE rapidly loses its ability to provide reinforcement due to softening/melting, while all PIBOMA thermoplastics maintain their ability to reinforce the rubber matrix.

**Figure 6:**
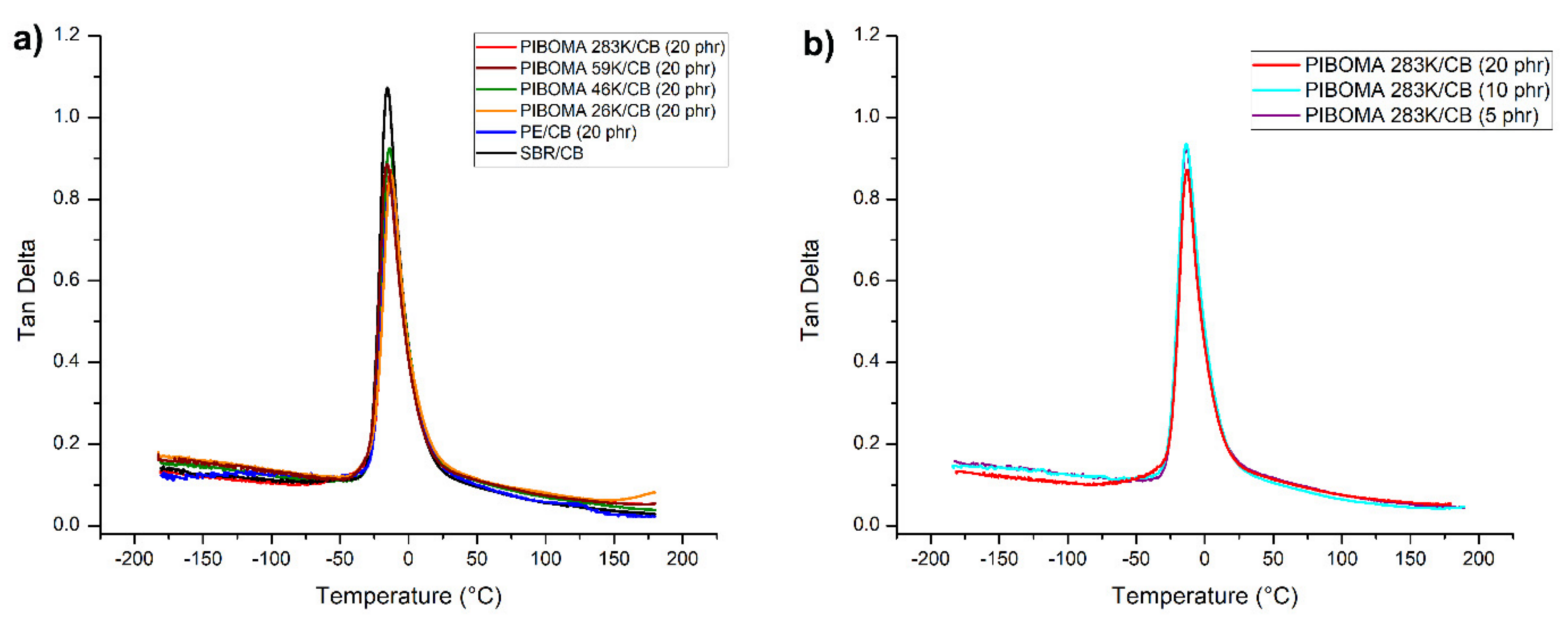
Tan δ as a function of temperature for vulcanized rubber compounds containing 50 phr of CB and and filled with 20 phr of PIBOMA 283 K and 20 phr of UHMWPE for comparison (**a**) and of those containing different amounts of PIBOMA 283 K (**b**).

As PIBOMA 283 K provided the highest degree of reinforcement over the largest range of temperatures, an additional study was made concerning the effects of its concentration on the mechanical behavior. As expected, storage modulus levels increased at all temperatures with increasing PIBOMA 283 K content (Figure 5b). A similar trend was observed for the temperature dependence of loss modulus (Figure 5c). Thus, the increase in the content of PIBOMA 283 K increases not only the dynamic stiffness (G′) but also the dynamic hysteresis (G″). This strongly suggests that especially in the temperature range above about 10 °C the PIBOMA acts in analogy to a reinforcing filler showing a systematic increase in storage and loss modulus through hydrodynamic reinforcement. Using a simple concept of hydrodynamic reinforcement with a constant hydrodynamic amplification factor for both, the storage and the loss modulus, it follows that tan δ is basically unchanged as observed experimentally in Figure 6, where the loss tangent curves for the tested thermoplastic-reinforced rubber compositions were found to superimpose on top of one another, with a single peak observed in all cases (Figure 6). No significant changes were observed in the loss tangent peak vs. the SBR/CB control, with the exception of the expected decrease in its strength in proportion to the amount of thermoplastic added (Figure 6b).

#### 3.3.3. Tensile Testing

**Figure 7:**
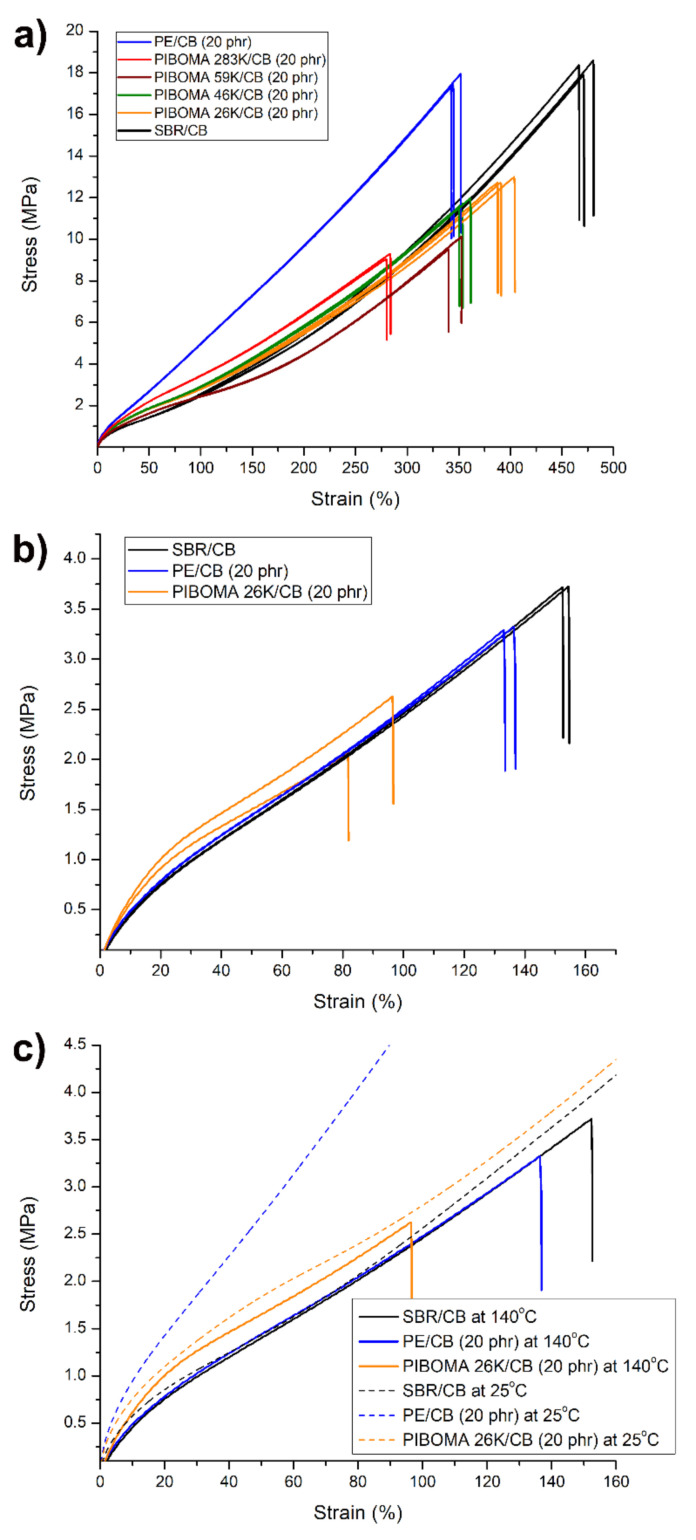
Stress–strain curves of vulcanized rubber compounds containing 50 phr of CB and filled either with 20 phr of PIBOMA having various molecular weights or with 20 phr of UHMWPE (at 25 (**a**) and 140 °C (**b**)). Overlaying of the stress-strain curves at 25 and 140 °C (**c**).

The stress–strain curves of vulcanized rubber compounds filled with 20 phr of PIBOMA having different molecular weights or filled with various concentrations of PIBOMA 283 K are shown in Figure 7 and Figure S7. The mechanical properties, tensile strength and elongation at break of the aforementioned vulcanized rubber compounds were compared with that of a vulcanized rubber compound filled with UHMWPE and with the carbon black reference compound (Table 3).

For vulcanized rubber compositions filled with 20 phr of PIBOMA, both tensile strength and elongation at break decreased with increasing PIBOMA molecular weight (Figure 7):

*σ*_t_ PIBOMA 26 K/CB (12.7 MPa) > *σ*_t_ PIBOMA 46 K/CB (11.7 MPa) > *σ*_t_ PIBOMA 59 K/CB (9.8 MPa) > *σ*_t_ PIBOMA 283 K/CB (9.1 MPa).

*ε*_t_ PIBOMA 26 K/CB (390%) > *ε*_t_ PIBOMA 46 K/CB (354%) > *ε*_t_ PIBOMA 59 K/CB (345%) > *ε*_t_ PIBOMA 283 K/CB (280%).

In addition, while the moduli of these vulcanized rubber compounds show typical strain-sensitive behavior, it is also evident that compound stiffness increases as PIBOMA molecular weight increases, consistent with what was previously observed via DMTA in the rubbery regime. One possibility here is that reductions in PIBOMA molecular weight favor uptake of small molecules (e.g. additives, waxes, etc.) by the PIBOMA domains through a diffusion mechanism, resulting in increased plasticization. It may also be that variations in the size of the PIBOMA domains with molecular weight and their changes in debonding behavior at the low strains play a role as well (see Section 3.3.4 and Section 3.3.5).

Additionally, when the concentration of the highest molecular weight PIBOMA 283 K was varied, the following trends in tensile strength and elongation at break were observed (Figure S7):

*σ*_t_ 5 phr of PIBOMA 283 K/CB (15.0 MPa) > *σ*_t_ 10 phr of PIBOMA 283 K/CB (14.7 MPa) > *σ*_t_ 20 phr of PIBOMA 283 K/CB (9.1 MPa).

ε_t_ 5 phr of PIBOMA 283 K/CB (365%) > *ε*_t_ 10 phr of PIBOMA 283 K/CB (330%) > *ε*_t_ 20 phr of PIBOMA 283 K/CB (280%).

As illustrated in Figure S7, increasing the content of PIBOMA 283 K resulted in the decrease of the tensile strength and elongation at break value and an increase in stiffness. This is consistent with literature reports indicating that PIBOMA is likely stiffer but weaker and more brittle than the cured CB-filled rubber studied here [50].

Comparing rubber compositions filled with PIBOMA and the control UHMWPE, it can be seen that the UHMWPE containing composition provides higher stiffness than all other rubber compositions and higher tensile strength than all but the thermoplastic-free composition (Figure 7). The fact that the UHMWPE-containing composition appears to be stiffer than all PIBOMA-containing compositions at all strain levels implies that the UHMWPE domains must be stiffer than the PIBOMA domains, in spite of the fact that literature reports indicate that PIBOMA should have a significantly higher modulus than UHMWPE (~1.6 GPa [50] vs. ~0.8–0.9 GPa [51]). This is consistent with the prior argument made concerning plasticization of the PIBOMA domains, as it is expected that an amorphous polymer such as PIBOMA would be more readily plasticized at room temperature than a highly crystalline polymer such as UHMWPE (X_c_ ~ 55–60% typically [51,52]). Additionally, it is noteworthy that the increased polarity of PIBOMA vs. UHMWPE might further favor the uptake of various polar rubber additives. This, in turn, could result in a reduction in the effective modulus of the PIBOMA domains present in the various PIBOMA-reinforced rubber compositions described here, thus explaining the decrease in stiffness vs. what might otherwise be expected. This logic also helps to explain the variations observed in elongation at break, with decreasing plasticization as PIBOMA molecular weight increases favoring greater stiffness but lower elongation in the associated vulcanized rubber compounds. The increase in tensile strength when the lowest molecular weights of PIBOMA are present is consistent with this explanation as well, as plasticization and interdiffusion at the PIBOMA-rubber interface are expected to enhance the ductility of the PIBOMA domains and possibly to increase the interfacial strength. At the same time, plasticization by additives alone is not expected to contribute to interfacial strength. This requires interdiffusion of the rubber itself, which is expected to be more favorable in the case of UHMWPE, given the much greater mismatch in polarity between PIBOMA and the SBR matrix. As such, a second explanation for the enhanced mechanical performance observed in the UHMWPE containing compounds at lower temperatures relates to the possibility of more effective interfacial stress transfer in these systems.

Highlighting the enhanced thermomechanical stability provided by PIBOMA in comparison with UHMWPE, the results of tensile testing at 140 °C are shown in Figure 7b. At this temperature it can be observed that UHMWPE provides no reinforcement at all, whereas PIBOMA continuous to provide modulus enhancements. The overlaying of the stress-strain curves obtained at 25 and at 140 °C clearly demonstrates (Figure 7c) the significant reduction in the slope of the curves related to the UHMWPE containing compositions, while the curves of PIBOMA 26 K/CB filled compositions remained practically unaltered.

#### 3.3.4. AFM

**Figure 8:**
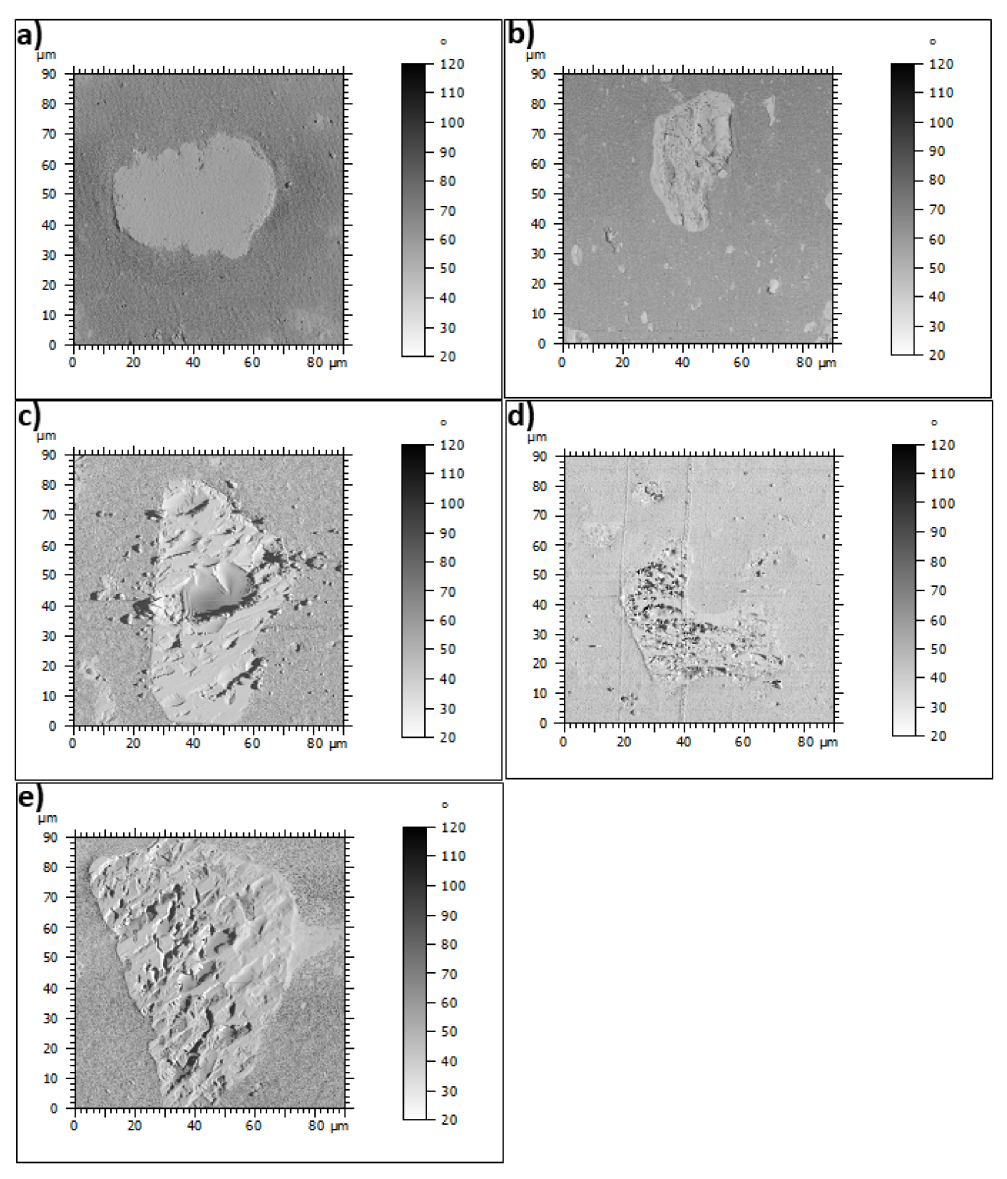
AFM tapping mode height images of vulcanized rubber compounds containing 50 phr of CB and filled with 20 phr of PE (**a**) and PIBOMA with molar masses *M*_n_ = 26,000 (**b**), 46,500 (**c**), 59,500 (**d**) and 283,000 g mol^−1^ (**e**), respectively.

The morphology of PIBOMA- and UHMWPE-filled vulcanized rubber compounds was investigated by AFM. Due to immiscibility between rubber and the thermoplastic polymers, phase separation was observed in macro-scale (Figure 8). While the rubber part (soft segments) represents dark regions, the thermoplastic part (hard segments) appears as bright areas. The rubber compositions filled with UHMWPE and PIBOMA in accordance to the size of their domains can be arranged in the following order:

UHMWPE (55 μm) ≈ PIBOMA 26 K (54 μm) < PIBOMA 46 K (45–80 μm) < PIBOMA 59 K (50–50 μm) < PIBOMA 283 K (65–90 μm).

As shown in Figure 8, the size of PIBOMA domains basically increased with increasing molar mass of PIBOMA.

#### 3.3.5. X-ray Computed Tomography

**Figure 9:**
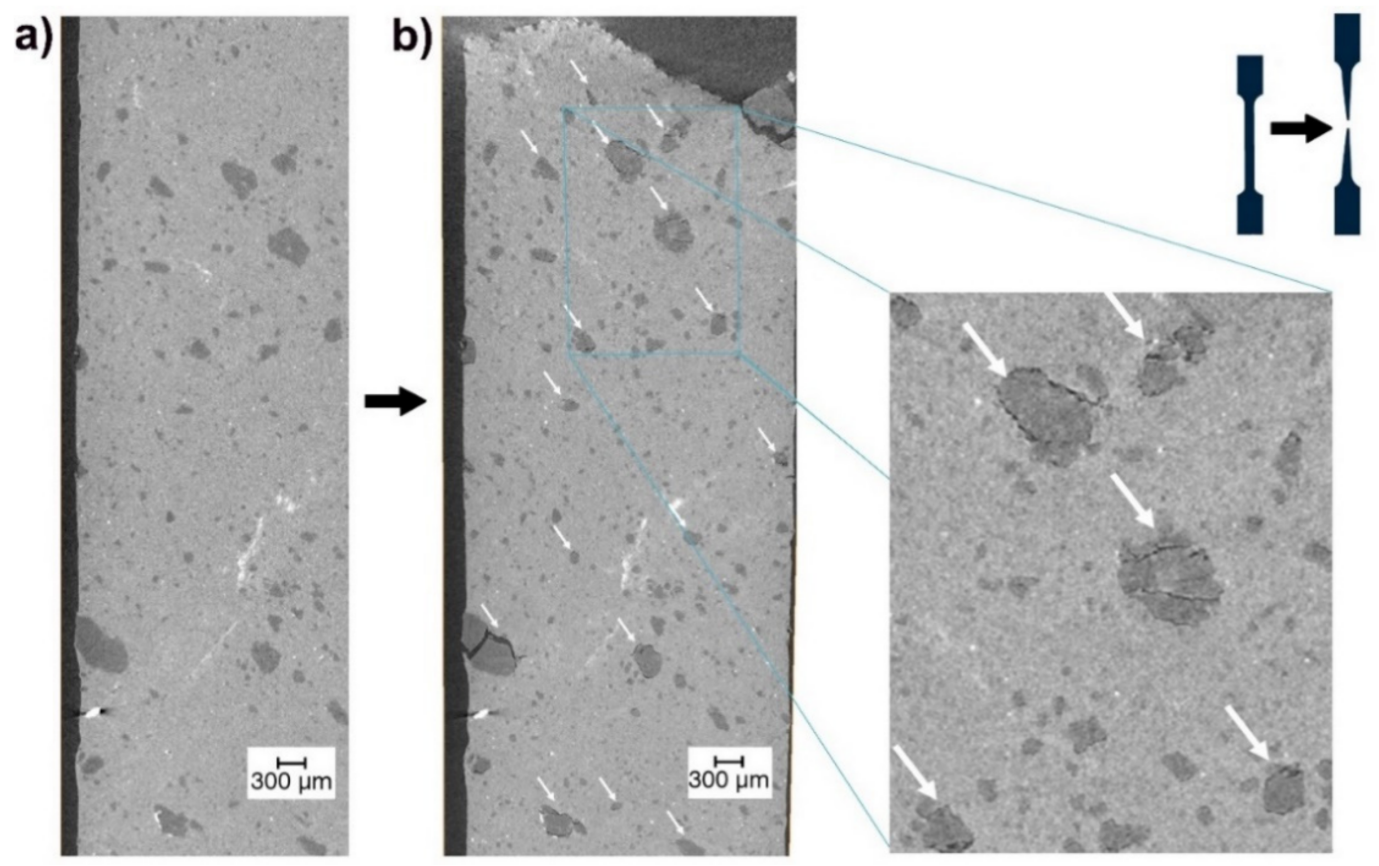
Representative X-ray computed tomography slices of vulcanized rubber compound filled with 50 phr of CB and 20 phr of PIBOMA (*M*_n_ = 283,000 g mol^−1^) before (**a**) and after its tensile testing (**b**). White arrows are for indication of the decohesion only.

The vulcanized rubber compound filled with PIBOMA 283 K was studied by X-ray computed tomography (CT) before and after tensile testing (Figure 9). Due to the difference in the density between the SBR and PIBOMA domains, large and small PIBOMA domains are observed in dark grey whereas the light grey corresponds to the rubber matrix. The size of the PIBOMA domains was ~90 μm, in good agreement with AFM imaging. The sample was subjected to monotonic tensile loading until fracture, and then scanned by X-ray CT analysis in the region where fracture took place. Post-mortem results revealed many micro-cracks both inside the PIBOMA domains and on their borders, suggesting a combination of internal rupture and interfacial debonding. The former is consistent with the very low fracture strain reported for PIBOMA in the literature [50], while the latter maybe related to the existence of a mechanically abrupt interface with minimal interdiffusion and no strong chemical bonds present (in other words, limited molecular interaction between the thermoplastic and the elastomer). Furthermore, the observation of numerous PIBOMA domains at the fracture surface supports the conclusion that debonding of the SBR rubber matrix from the PIBOMA domains is an important rupture mechanism in these materials (Figure 9).

## 4. Conclusions

The conclusions stated herein are derived from, and applicable to, the specific parameters implemented for this unique work. PIBOMA, a high *T*_g_ thermoplastic, is for the first time evaluated as a reinforcing polymer for vulcanized rubber compounds. For this purpose, a set of PIBOMAs with *M*_n_ = 26,000 ÷ 59,500 g mol^−1^ and low dispersity (*M*_w_/*M*_n_ < 1.2) were synthesized via RAFT polymerization. In order to enlarge the range of molar masses studied, an additional PIBOMA sample with *M*_n_ = 283,000 g mol^−1^ was prepared by free radical polymerization. The structure and purity of these polymers was confirmed by IR and NMR spectroscopy, while the study of their properties revealed that they possess good thermal stability (*T*_onset_ up to 230 °C) and high glass transition temperatures (*T*_g_ ~ 197–201 °C).

The synthesized PIBOMA samples were further tested as polymeric fillers in the preparation of vulcanized rubber compositions based on poly(styrene butadiene rubber) (SBR) and containing 50 phr of carbon black. The effect of the content and molar mass of PIBOMA on the properties of the filled rubber compositions was discussed in detail and compared with CB control compound. In MDR measurements all samples filled with CB and PIBOMA displayed higher Δ*S* values relative to the CB control compound, irrespective of PIBOMA molecular weight, confirming the high temperature reinforcing effect of PIBOMA. Similar effects were observed in DMTA, where for vulcanized rubbers in the range of ~30–125 °C, the reinforcing efficiency of low molecular weight PIBOMAs was competitive with that of UHMWPE and PIBOMA 283 K exceeded the performance of UHMWPE throughout this range. At temperatures above ~125 °C the UHMWPE rapidly loses its ability to provide reinforcement due to softening/melting, whereas all PIBOMA resins maintain their capacity to reinforce the rubber matrix up to 180 °C.

Finally, with respect to the tensile testing results, it was observed that, for rubber compositions filled with 20 phr of PIBOMA, both tensile strength and elongation at break decreased with increasing PIBOMA molecular weight. Likewise, increasing PIBOMA 283 K content also gave decreases in tensile strength and elongation at break in tandem with increased stiffness. To better understand this result, AFM and X-ray computed tomography were used to characterize rubber compositions before and after tensile testing. The AFM results revealed that the size of the PIBOMA domains in the rubber compositions increased from 65 to 90 μm as the PIBOMA molar mass increased from 26,000 to 283,000 g mol^−1^. Moreover, X-ray computed tomography revealed that fracture arose from either debonding at the rubber-PIBOMA interface at high strains, with cracks observed where the PIBOMA domains were concentrated or from the cracks happened inside the PIBOMA domains. This indicates that PIBOMA is likely stiffer but weaker and more brittle than the cured CB-filled rubber. It was further observed that UHMWPE provided higher stiffness than PIBOMA in tensile testing at room temperature as well. However, this is not inconsistent with the DMTA data, which shows that UHMWPE does provide the highest level of reinforcement under these conditions. In contrast to PIBOMA containing compositions, the size of the UHMWPE domains is consistently smaller (~55 μm), and the observation of higher tensile strength in the UHMWPE containing compounds implies a reduced tendency for interfacial debonding, consistent with the possibility that the UHMWPE undergoes partial melting during rubber compound processing and curing. This, coupled with its greater anticipated compatibility with the relatively non-polar rubber matrix and its susceptibility to radical attack, may tend to favor the formation of a stronger interface in the UHMWPE case under the parameters of this work. Nevertheless, the heat generation typically observed in high temperature vulcanized rubber compound applications results in a need for stiffness at temperatures significantly higher than room temperature, where our high temperature stress–strain and viscoelastic data tends to show that the performance of UHMWPE may suffer more than PIBOMA. In this specific context, beyond the high temperature reinforcement reported here, we emphasize the potential of PIBOMA to provide additional levels of performance following potential, new improvements in interfacial strength and process optimization in the future.

## Figures and Tables

**Table 1:** Examples of the thermoplastics considered for the reinforcement of rubber compositions in various applications.

Entry	Thermoplastic		*T*_g_ (°C)	*T*_m_ (°C)	Ref.
	Name	Structure			
1	UHMWPE	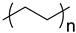	−100 ÷ −125	135	[35,36,37]
2	Syndiotactic 1,2-polybutadiene/1,4-cis polybutadiene (12:88 by wt.)	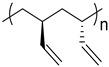	−104 (for cis-PBD)	200 (for syndio-PBD)	[22]
3	Syndiotactic 1,2-polybutadiene		-	126	[21]
4	Syndiotactic 1,2-polybutadiene		-	200 ÷ 201	[34]
5	Isotactic polypropylene	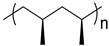	-	152	[28,29]
6	Syndiotactic polystyrene	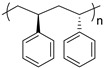	98	262	[26]
7	Polystyrene *M*_n_ = 3.5 × 10^5^ g mol^−1^	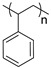	106	-	[25]
8	PMMA	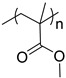	119	-	[27]
9	Poly(butadiene-r-(2-ethylhexyl methacrylate)) 54.5:45.5 by mole *M*_n_ = 2.5 × 10^5^ g mol^−1^	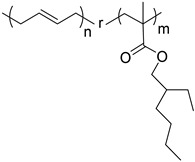	−53	-	[38]
10	Poly(butadiene-r-(glycidyl methacrylate)-r-styrene)) 86:5:9 by mole	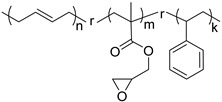	−59	-	[38]
11	Poly((2-ethylhexyl)-r-(*n*-octyl methacrylate)) ~50:50 by mole *M*_n_ = 7.1 × 10^4^ g mol^−1^	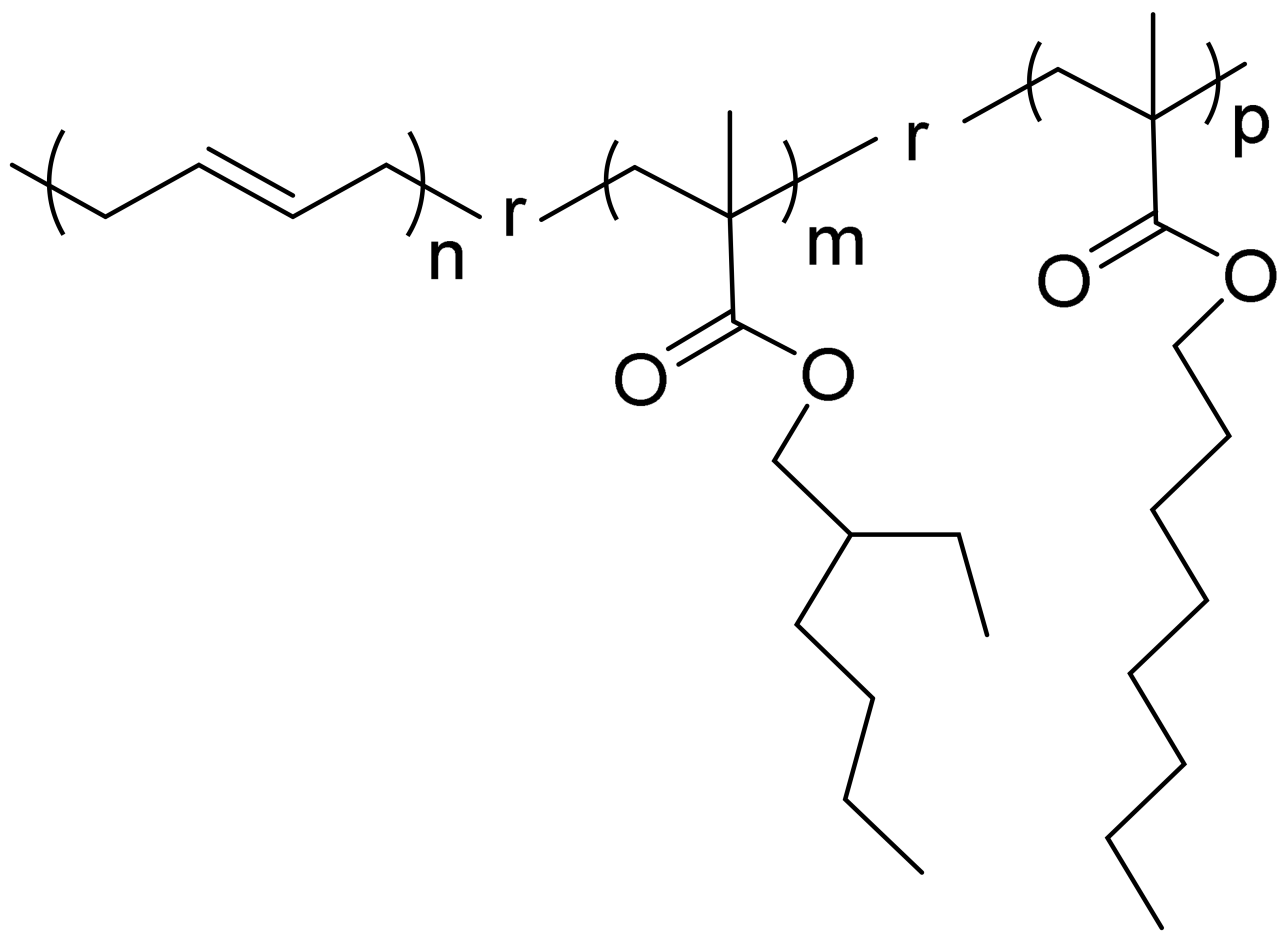	−50	-	[38]
12	Polyamide-6	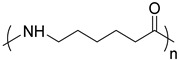	-	220	[31]
13	Poly(ethylene terephthalate)	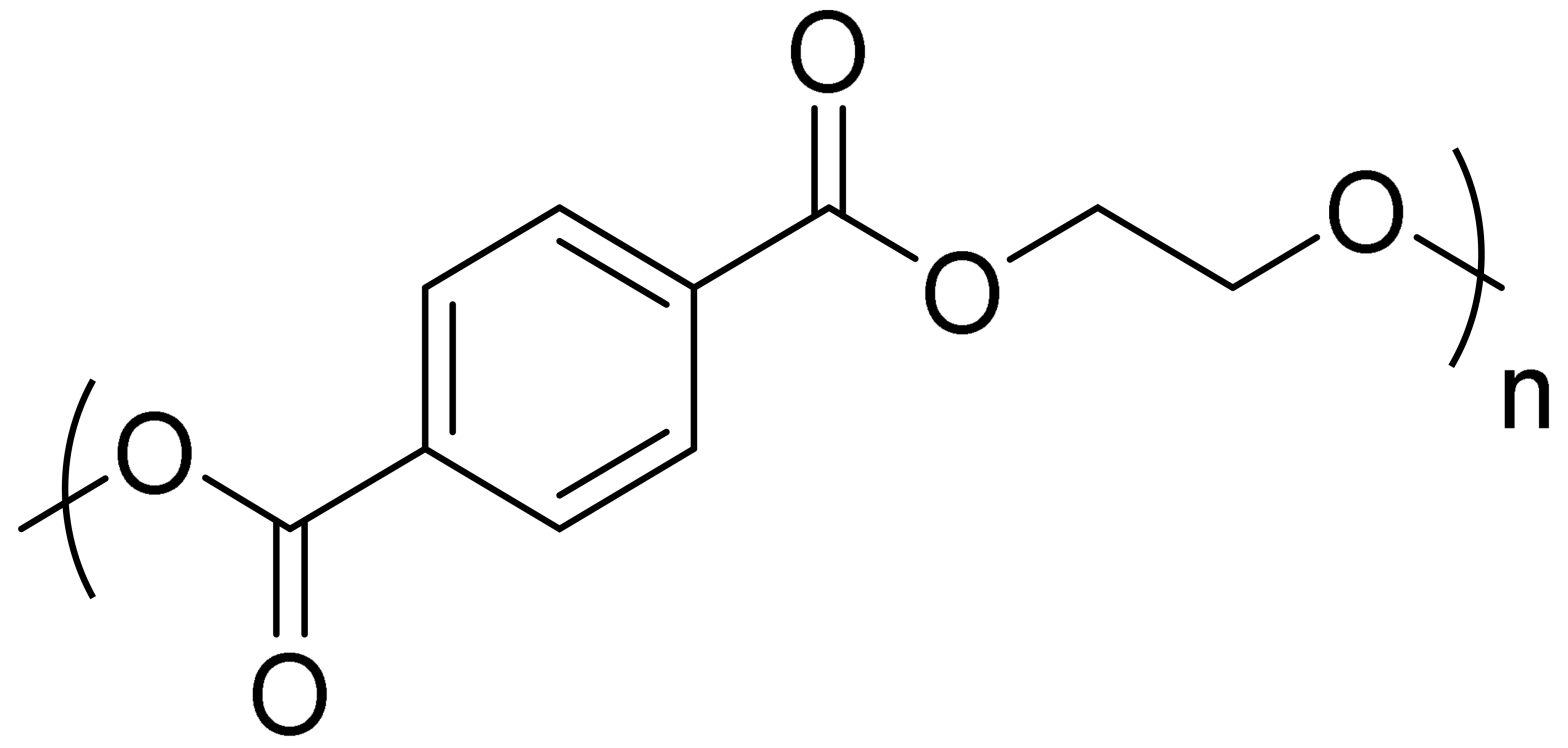	76	253	[30]
14	Poly-*p*-phenylene terephtalamide	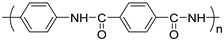	327	-	[40]
15	Poly-*m*-phenylene isophtalamide	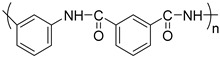	285	-	[40]
16	Bisphenol A polycarbonate	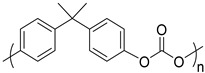	147	280	[33]

**Table 2:** Formulation of the rubber compounds.

Ingredients	Amounts (Parts per Hundred Rubber, phr)
**Stage NP**	
Styrene Butadiene rubber (SBR)	100
Thermoplastic polymer (PIBOMA or UHMWPE)	20
Carbon black (CB)	50
*N*-(1,3-dimethylbutyl)-*N*′-phenyl-p-phenylenediamine (6-PPD, antioxidant)	2.5
Stearic acid (activator)	2
TDAE oil	4
Wax 1 (microcrystalline wax)	1
Wax 2 (refined paraffin wax)	0.5
**Stage PR**	
Zinc Oxide (activator)	2.5
Sulfur (curative)	1.5
*N*-cyclohexyl-2-benzothiazolesulfenamide (CBS, accelerator)	1.8
Diphenylguanidine (accelerator)	0.5

**Table 3:** Selected properties of thermoplastic polymers and vulcanized rubber compounds reinforced with them (the loading of thermoplastic in rubber composition was always kept at 20 phr level).

Entry	Neat Thermoplastic					Vulcanized Rubber Composition			
		*M*_n_ (g mol^−1^) ^1^	*M*_w_/ *M*_n_ ^1^	*T*_g_ (°C) ^2^	*T*_onset_ (°C) ^3^	*σ*_t_ (MPa) ^4^	*ε*_t_ (%) ^5^	*S*’max (dNm) ^6^	(Δ*S*) (dNm) ^7^
1	PIBOMA 26 K	26,000	1.1	197	225	12.7 ± 0.2	394 ± 9	20.0	18.1
2	PIBOMA 46 K	46,400	1.2	197	230	11.7 ± 0.2	356 ± 6	20.6	18.8
3	PIBOMA 59 K	59,400	1.2	201	225	9.8 ± 0.3	346 ± 6	21.4	19.5
4	PIBOMA 283 K	283,000	1.9	199	225	9.1 ± 0.2	280 ± 4	21.8	19.7
5	PE	4.7 × 10^6^	-	135 ^8^ (142 ^9^)	210	17.5 ± 0.6	348 ± 6	17.7	15.4
6 ^10^	-	-	-	-	-	18.3 ± 0.3	473 ± 8	16.9	15.2

^1^ By GPC in THF at 40 °C (calibration with PMMA standards). ^2^ By TMA. ^3^ Onset weight loss temperature by TGA on air. ^4^ Tensile strength at 25 °C. ^5^ Elongation at break at 25 °C. ^6^ Maximum torque by MDR. ^7^ Torque increase by MDR. ^8^ *T*_m_ by TMA. ^9^ *T*_m_ by DSC. ^10^ The vulcanized SBR/CB control is provided for comparison.

## Supplementary Materials

The following are available online at https://www.mdpi.com/article/10.3390/polym13101626/s1. Table S1: Results on free radical polymerization of IBOMA. Table S2: Solubility of PIBOMA. Figure S1: GPC traces of PIBOMA samples. Figures S2 and S3: ^1^H and ^13^C NMR of PIBOMA 26 K. Figure S4: FT-IR spectrum of PIBOMA 26 K. Figures S5 and S6: TMA and TGA traces of PIBOMA having different molar masses. Figure S7: Stress–strain curves of SBR compounds containing 50 phr of CB and filled with various content of PIBOMA 283 K. Figure S8: Specimen dimensions used in tensile testing.
