# Supplementary material for: Reinforcement of Styrene Butadiene Rubber Employing Poly(isobornyl methacrylate) (PIBOMA) as High Tg Thermoplastic Polymer
Source: Polymers (Basel). 2021 May 17;13(10):1626. doi: 10.3390/polym13101626 (PMC8157114; doi:10.3390/polym13101626)
Supplement: Supplementary file 1 [file polymers-13-01626-s001.zip › polymers-1217944-supplementary.pdf]

Supplementary Materials

Reinforcement of Styrene Butadiene Rubber Employing Poly(isobornyl methacrylate) (PIBOMA) as High T_g Thermoplastic Polymer

Abdullah Gunaydin[1,2], Clément Mugemana[1], Patrick Grysan[1], Carlos Eloy Federico[1], Reiner Dieden[1], Daniel F. Schmidt[1], Stephan Westermann[1], Marc Weydert[3] and Alexander S. Shaplov[1,*]

[1] Luxembourg Institute of Science and Technology, Materials Research and Technology Department, 5 Avenue des Hauts-Fourneaux, L-4362 Esch-sur-Alzette, Luxembourg

[2] Department of Physics and Materials Science, University of Luxembourg, 2, Avenue de l'Université, L-4365 Esch-sur-Alzette, Luxembourg

[3] Goodyear Innovation Center Luxembourg, L-7750 Colmar-Berg, Luxembourg

Table S1. Free radical polymerization of IBOMA (reaction conditions: [AIBN] = 1.0 wt%, duration: 6h, [IBOMA] = 50 wt%).

Entry	Solvent	Initiator	T [°C]	M_n (g mol^{-1})[a]	M_w/M_n[a]	Yield [%]
**1**[b]	DMF	AIBN	60	715800[c]<br>59250	1.6<br>2.2	29
**2**	Toluene	AIBN	60	73600	2.4	44
**3**	THF	AIBN	60	59500	2.2	74
**4**	Cyclohexane	AIBN	60	151800	2.3	41
**5**	1,1,2-TCA	AIBN	60	68400	2.7	71
**6**	1,1,2-TCA	ACHN	88	43600	2.4	77
**7**[d]	1,1,2-TCA	ACVA	69	89000	2.1	75
**8**	1,1,2-TCA	VAm-110	110	82900	3.1	80
**9**[e]	1,1,2-TCA	VAm-110	110	283000	1.9	70
**10**	Cyclohexane	ACHN	88	74300	2.6	49

[a]Molecular weight and M_w/M_n determined by GPC in THF at 40°C with calibration by PMMA standards. [b] The precipitation of PIBOMA was observed. [c] Bimodal distribution. [d]ACVA was found to be insoluble in 1,1,2-TCA. [e]Concentration of [AIBN] = 0.5 wt%.

	Solvents [b]											
Polymer	DCM	Chloroform	TCA	THF	DMF	DMAc	Cyclohexane	Toluene	NMP	Et_2O	ACN	MeOH	Acetone
PIBOMA	+	+	+	+	+[t]	+	+[t]	+	-	+	-	-	-

Table S2. Solubility of PIBOMA[a]

[a] Solubility: soluble at room temperature (+), soluble at 80°C (+[t]), insoluble even on heating (-). [b] DCM - dichloromethane; TCA - 1,1,1-trichloroethane; THF- tetrahydrofuran; DMF - N,N-dimethylformamide; DMSO - dimethyl sulfoxide; DMAc - N,N-dimethylacetamide; NMP - N-methyl-2-pyrrolidone; Et_2O - diethyl ether; ACN - acetonitrile; MeOH - methanol.

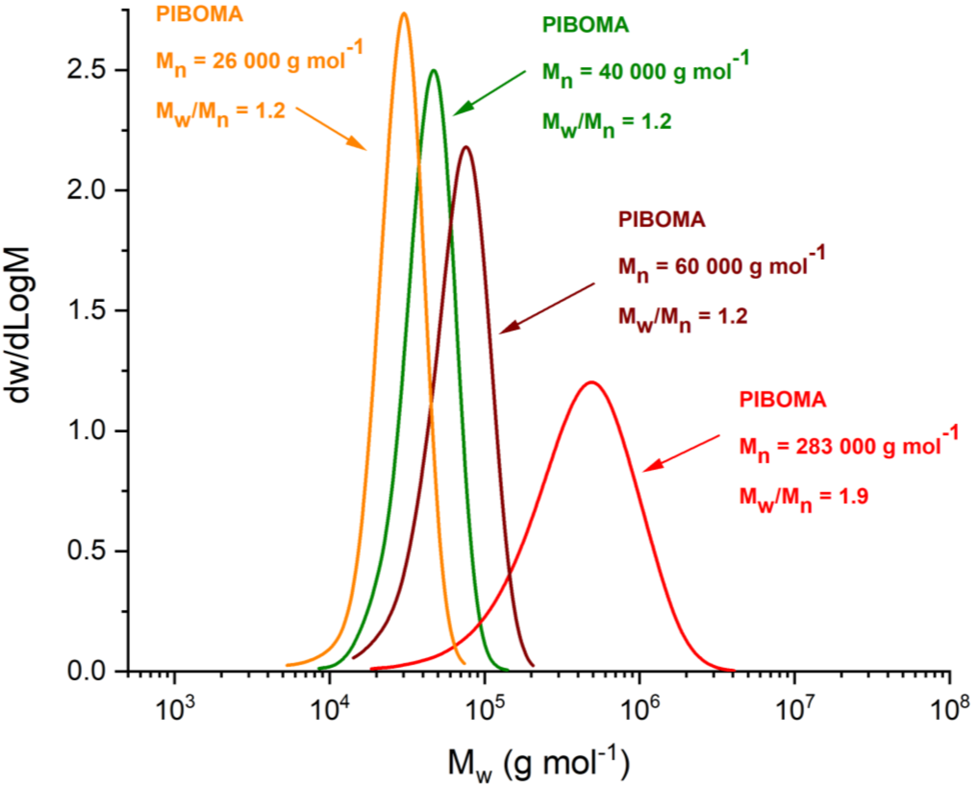

Figure S1. GPC traces of PIBOMA samples obtained via RAFT and free radical polymerization.

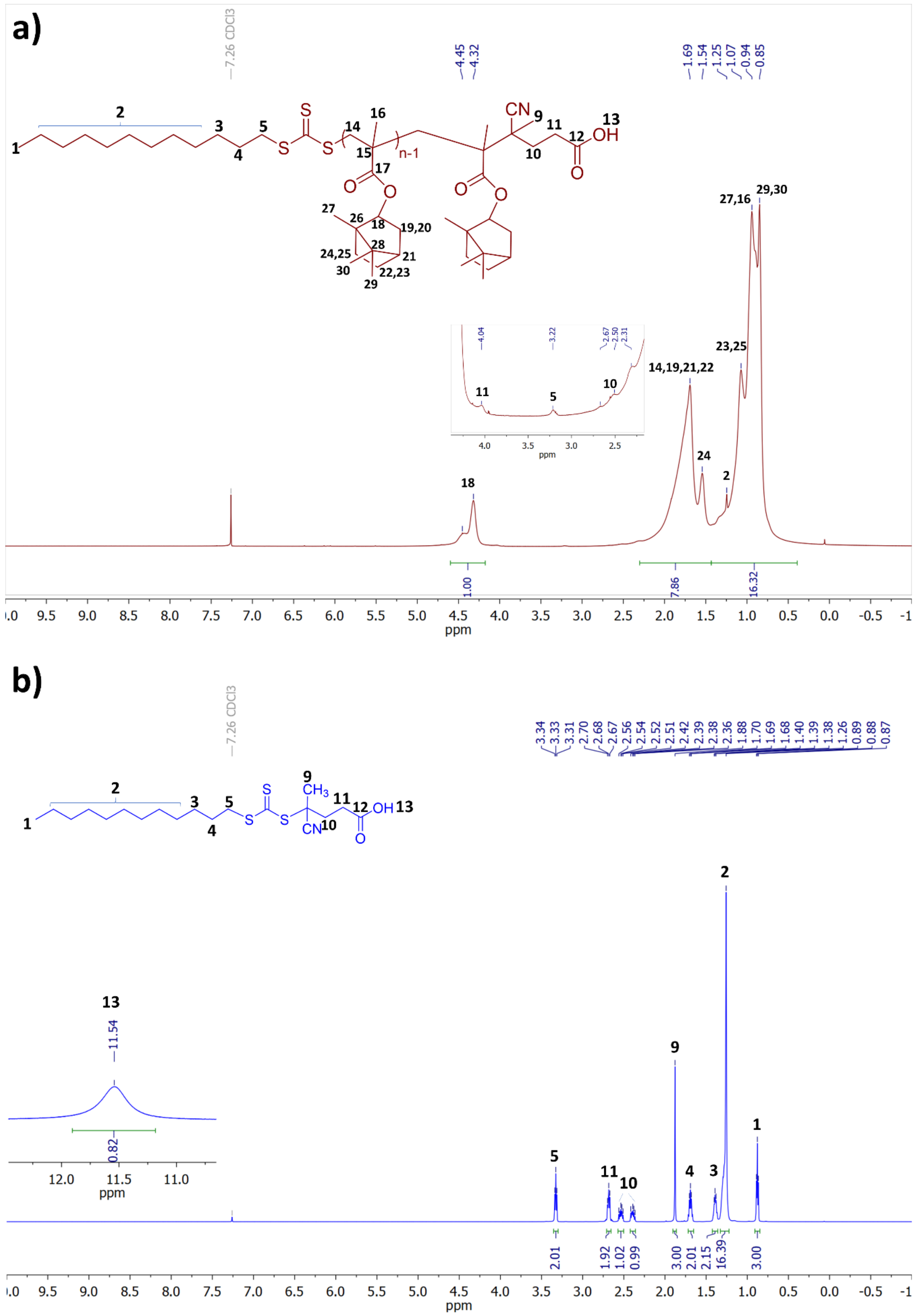

Figure S2. ^{1}H NMR of PIBOMA 26K (a) and CDTPA RAFT agent (b) (25 °C, $CDCl_3$).

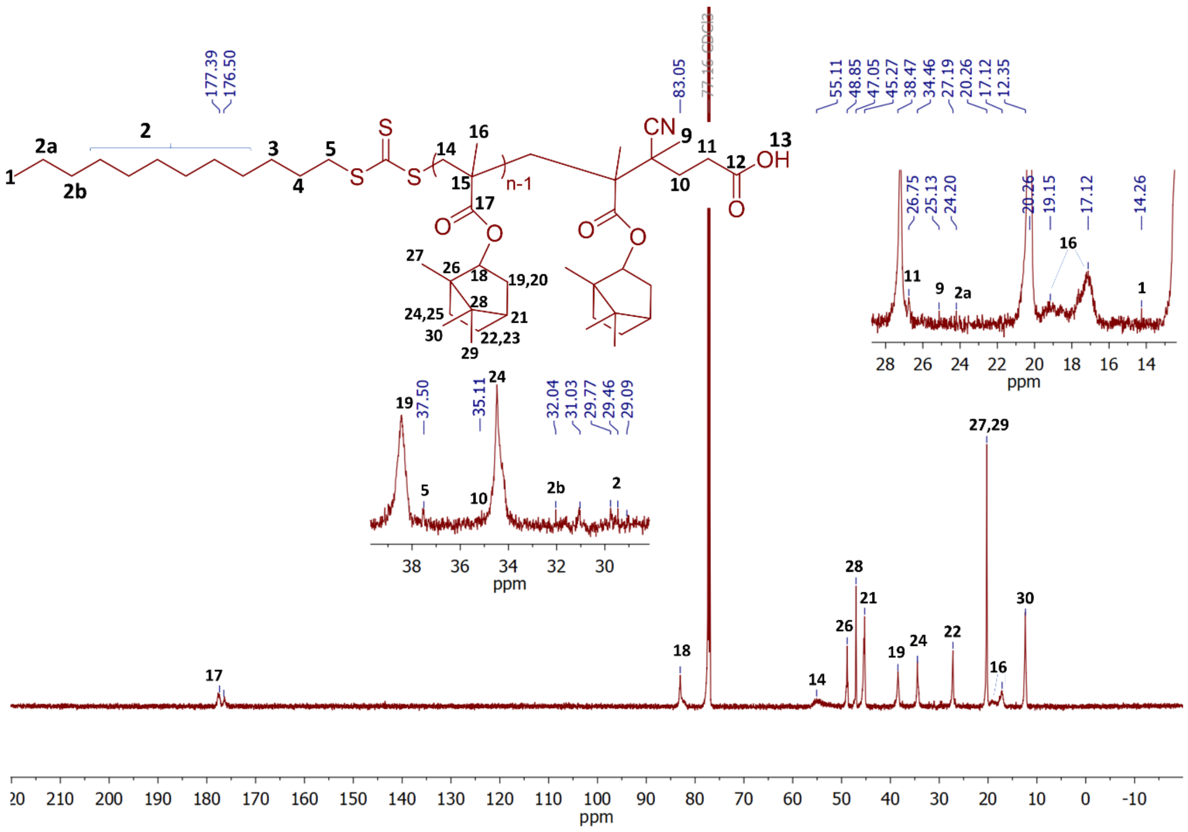

Figure S3. ^{13}C NMR of PIBOMA 26K (25 °C, $CDCl_3$).

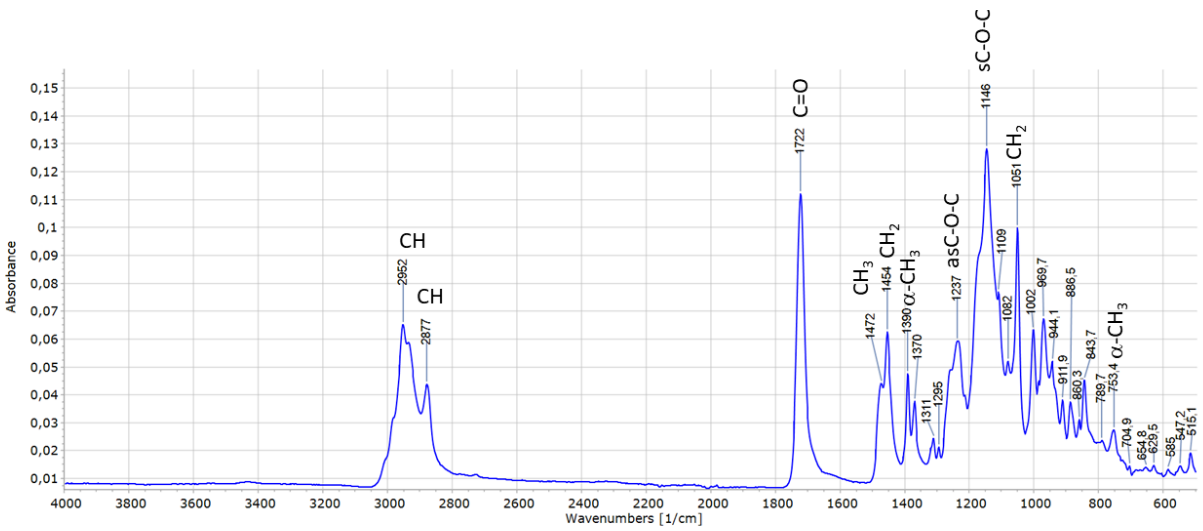

Figure S4. FT-IR spectrum of PIBOMA 26K.

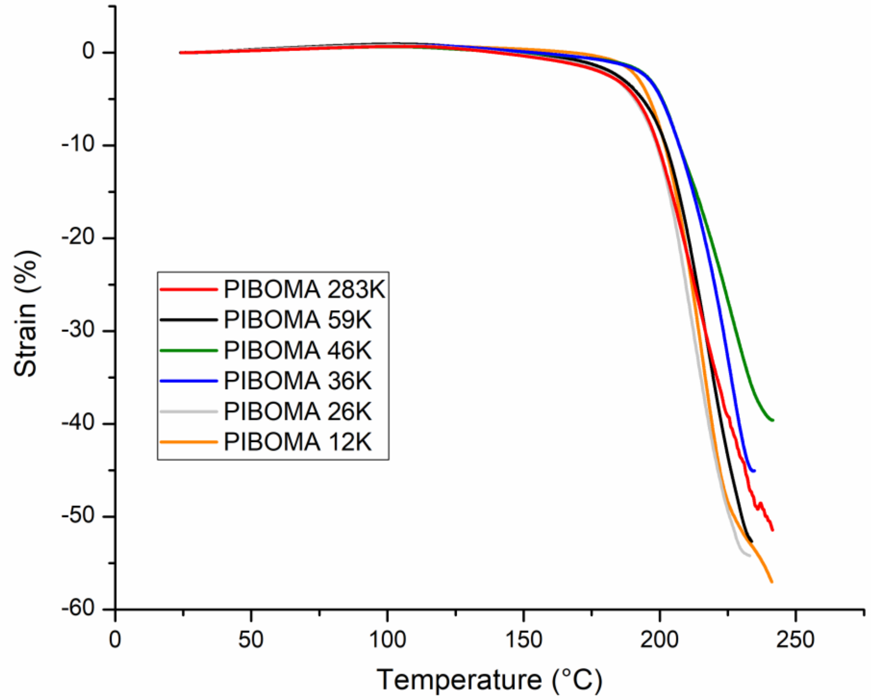

Figure S5. TMA traces of PIBOMA having different molar masses.

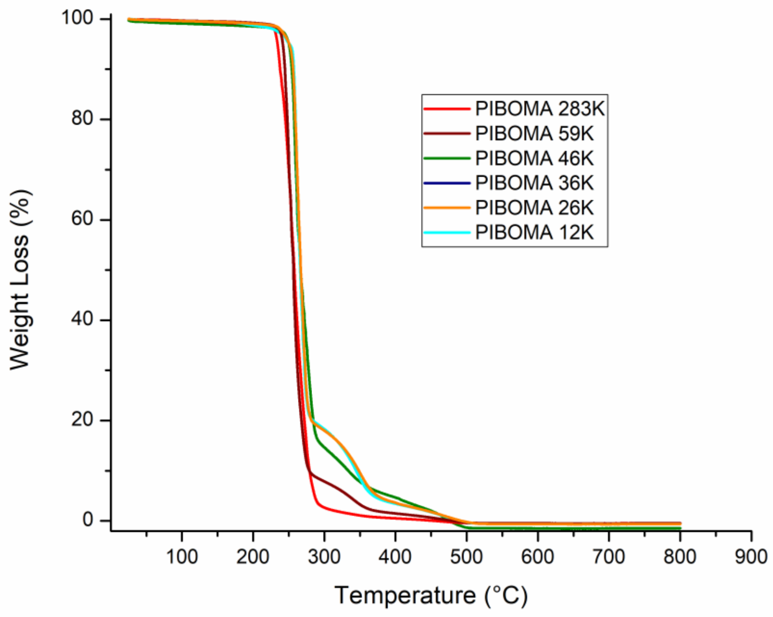

Figure S6. TGA traces of PIBOMA having different molar masses (TGA was performed in air with a heating rate of 5°C min^{-1}).

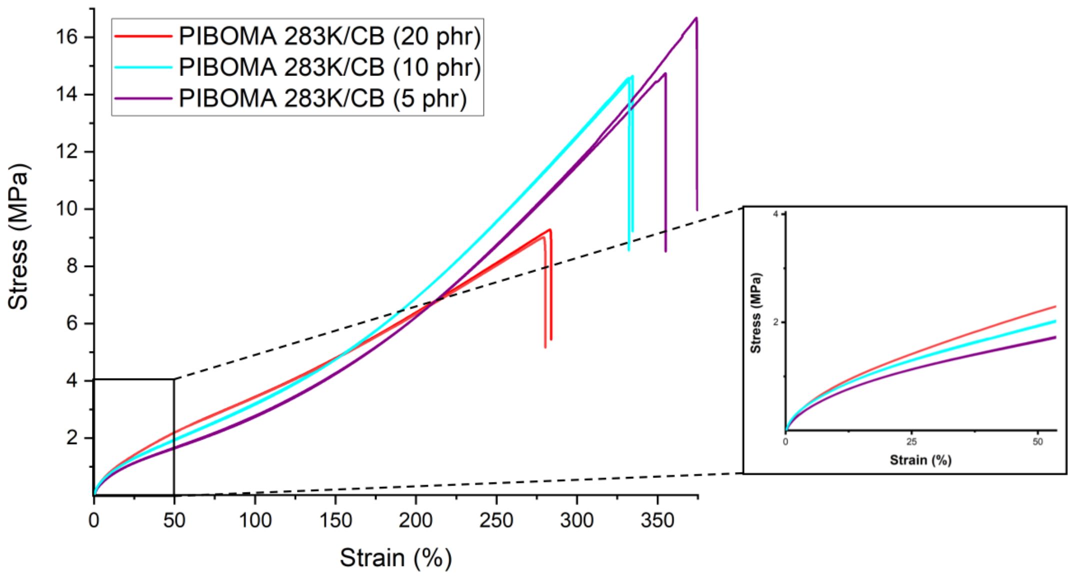

Figure S7. Stress–strain curves of SBR compounds containing 50 phr of CB and filled with increasing content of PIBOMA283K.

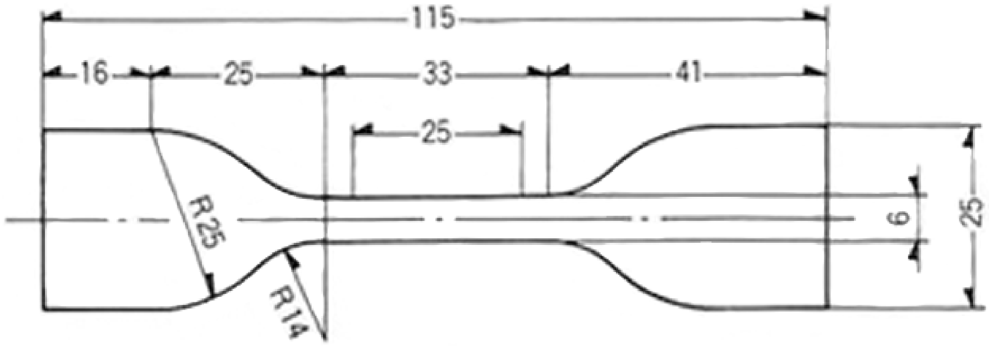

Figure S8. Specimen dimensions used in tensile testing (correspond to Type V in ASTM D638-14).
